# Alien plant fitness is limited by functional trade‐offs rather than a long‐term increase in competitive effects of native communities

**DOI:** 10.1002/ece3.10468

**Published:** 2023-09-01

**Authors:** Marco R. Brendel, Frank M. Schurr, Christine S. Sheppard

**Affiliations:** ^1^ Institute of Landscape and Plant Ecology University of Hohenheim Stuttgart Germany; ^2^ Division of Conservation in Agriculture German Federal Agency for Nature Conservation Bonn Germany

**Keywords:** biological invasions, biotic resistance, evolution of biotic interactions, interspecific competition with native communities, residence time, trait‐demography relationships

## Abstract

Alien plants experience novel abiotic conditions and interactions with native communities in the introduced area. Intra‐ and interspecific selection on functional traits in the new environment may lead to increased population growth with time since introduction (residence time). However, selection regimes might differ depending on the invaded habitat. Additionally, in high‐competition habitats, a build‐up of biotic resistance of native species due to accumulation of eco‐evolutionary experience to aliens over time may limit invasion success. We tested if the effect of functional traits and the population dynamics of aliens depends on interspecific competition with native plant communities. We conducted a multi‐species experiment with 40 annual Asteraceae that differ in residence time in Germany. We followed their population growth in monocultures and in interspecific competition with an experienced native community (varying co‐existence times between focals and community). To more robustly test our findings, we used a naïve community that never co‐existed with the focals. We found that high seed mass decreased population growth in monocultures but tended to increase population growth under high interspecific competition. We found no evidence for a build‐up of competition‐mediated biotic resistance by the experienced community over time. Instead, population growth of the focal species was similarly inhibited by the experienced and naïve community. By comparing the effect of experienced and naïve communities on population dynamics over 2 years across a large set of species with a high variation in functional traits and residence time, this study advances the understanding of the long‐term dynamics of plant invasions. In our study system, population growth of alien species was not limited by an increase of competitive effects by native communities (one aspect of biotic resistance) over time. Instead, invasion success of alien plants may be limited because initial spread in low‐competition habitats requires different traits than establishment in high‐competition habitats.

## Graphical abstract

The fitness of alien plants in their new area should be determined by functional traits that are related to invasiveness and effects of competition by native plant communities that may depend on their eco‐evolutionary experience and thus interact with residence time of the invader. In this context, the role of traits for fitness is likely to change depending on the habitat that is being invaded and may thus be determined by competition.

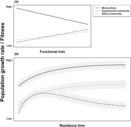

## 1 INTRODUCTION

The success of alien species is commonly studied in terms of their invasiveness and community invasibility (Alpert et al., 2000; Milbau et al., 2003). However, the success of aliens may depend on the environmental conditions in the new range. Moreover, community invasibility may depend on the time native species had to gain eco‐evolutionary experience of the invader. Thus, the combined effects of the new biotic and abiotic environment directly affect fitness and consequently impose selection on alien species. This selection can operate both between species (causing extinction of poorly adapted species and persistence of better adapted ones; Vellend, 2016) or it can operate within species (causing better adapted genotypes to increase in frequency). For instance, due to poor adaptation to the abiotic environment, alien fitness may initially be constrained in the new area (Brendel et al., 2021; Colautti et al., 2010). Alien fitness may then potentially increase with residence time due to the joint effect of intra‐ and interspecific selection exerted by the new abiotic environment (Brendel et al., 2021). However, how a gain in eco‐evolutionary experience of native species affects alien fitness, has only rarely been tested.

Higher fitness may result from functional trait values that better reflect adaptations to the new environment. Functional traits are defined as morphological, physiological, or phenological characteristics of an organism which impact fitness indirectly via their effects on demography (Violle et al., 2007). Functional traits that increase invasiveness are for instance low seed mass (that is related to a high reproductive output and dispersal rate), high specific leaf area (SLA, related to fast growth) and increased height (Catford et al., 2019; Conti et al., 2018; van Kleunen et al., 2010). The latter might evolve as a result of enemy release and a higher investment in competition than defense (evolution of increased competitive ability; Blossey & Nötzold, 1995). However, the role of functional traits that favor invasiveness might change depending on the habitat type being invaded (Alpert et al., 2000; Dietz & Edwards, 2006; Müller‐Schärer & Steinger, 2004).

Indeed, in various habitats, alien plants may experience differential selection regimes on functional traits related to population growth, dispersal, and competitive ability that in turn determine invasion success in the new area (Dietz & Edwards, 2006; Richardson & Pyšek, 2012; Theoharides & Dukes, 2007). In particular, ruderal habitats with low interspecific competition, where alien plants can form monospecific stands and intraspecific competition thus dominates, select for species and genotypes with low individual seed mass and high reproductive capacity (Grime, 2001) that increase their abundance more rapidly than others (Dietz & Edwards, 2006). Seed mass shows an inverse relationship with per capita fecundity (Moles et al., 2004; Turnbull et al., 1999) and leads to increased fecundity of small‐seeded species (Henery & Westoby, 2001). In low‐density monocultures, alien annual plants with low seed mass showed the highest intrinsic population growth rate (Brendel et al., 2021). Accordingly, as expected from intra‐ and interspecific selection, with increasing residence time seed mass converged toward low values (Brendel et al., 2021).

On the other hand, in semi‐natural habitats (i.e., remnants of habitats created by extensive, traditional farming, or restored natural vegetation for instance on land abandoned from agriculture; Pigott & Walters, 1954) with high interspecific competition, selection might favor traits related to enhanced competitive ability (Dietz & Edwards, 2006), such as increased height (Westoby, 1998) and high seed mass (Moles & Westoby, 2004). Under strong interspecific competition, a high investment in reproduction is disadvantageous (Lachmuth et al., 2011). Thus, it seems reasonable to expect that a trait‐mediated trade‐off between rapid population growth in low‐competition habitats and high competitive ability in competitive habitats limits alien plant invasions. Indeed, such trade‐offs strongly contribute to species co‐existence in native communities (Maron et al., 2021) and are consistently found on a global scale (Kunstler et al., 2016). However, direct links between functional traits of alien plants and population growth rates (representing population fitness, as opposed to considering only individual demographic rates or performance proxies) in different environments are so far lacking, although being vital for robust predictions of population dynamics (Laughlin et al., 2020).

Invasion success may in some instances also depend more strongly on characteristics of native communities than on the traits and competitive ability of the invader itself (Catford et al., 2019; Perry et al., 2009). In particular, competition, parasitism, and predation/herbivory can all mediate “biotic resistance” of the native community to the invader (Alpert, 2006; Levine et al., 2004). Biotic resistance can either completely repel invaders, or, as found to be more likely, reduce invasion success (Levine et al., 2004). As for competition‐mediated biotic resistance, native plant species are expected to gain eco‐evolutionary experience to the presence of the invader and might thus increase their competitive effects on the invader over time (Saul et al., 2013; Strauss et al., 2006). Whether a build‐up in such competition‐mediated biotic resistance decreases the fitness of alien species over time has rarely been tested (but see Sheppard & Schurr, 2019; Germain et al., 2020), although it is key to gain a more mechanistic understanding of the drivers of such a natural barrier to invasions (Gallien & Carboni, 2017).

In a recent study on biotic resistance of a native community to alien plants with varying residence times, Sheppard and Schurr (2019) found that the native community suppressed species of longer residence time relatively more. However, it is possible that this finding results from potentially confounding effects of species characteristics that may co‐vary with time since introduction and determine invasion success in interspecific competition. Specifically, alien species with longer residence times (i.e., archaeophytes, defined as plant species that were introduced into Europe prior to AD 1500) and natives may per se be less competitive than species that have been introduced only recently (i.e., neophytes; Sheppard & Schurr, 2019). For instance, archaeophytes, many of which are agricultural weeds and suppressed by crops during the cultivation period, are adapted to a release from competition with agricultural crops late in the growing season (Knapp & Kühn, 2012). In contrast, neophytes are commonly thought to be highly competitive. To conclusively disentangle such an inherent competitive ability from an evolutionary build‐up of biotic resistance, the following steps are required: (a) population growth of alien plants needs to be investigated and linked to invader functional traits (Laughlin et al., 2020) in different competitive regimes (i.e., habitats of low vs. high competition; Dietz & Edwards, 2006) and (b) biotic resistance needs to be studied in an experienced native community whereby the length of potential co‐existence time between aliens and natives varies (Sheppard & Schurr, 2019) as well as (c) in a naïve community that never co‐existed with the introduced species (Germain et al., 2020). To our knowledge, these three aspects have not yet been integrated into one experiment covering a large number of species and a wide range of functional traits and residence times.

**FIGURE 1:**
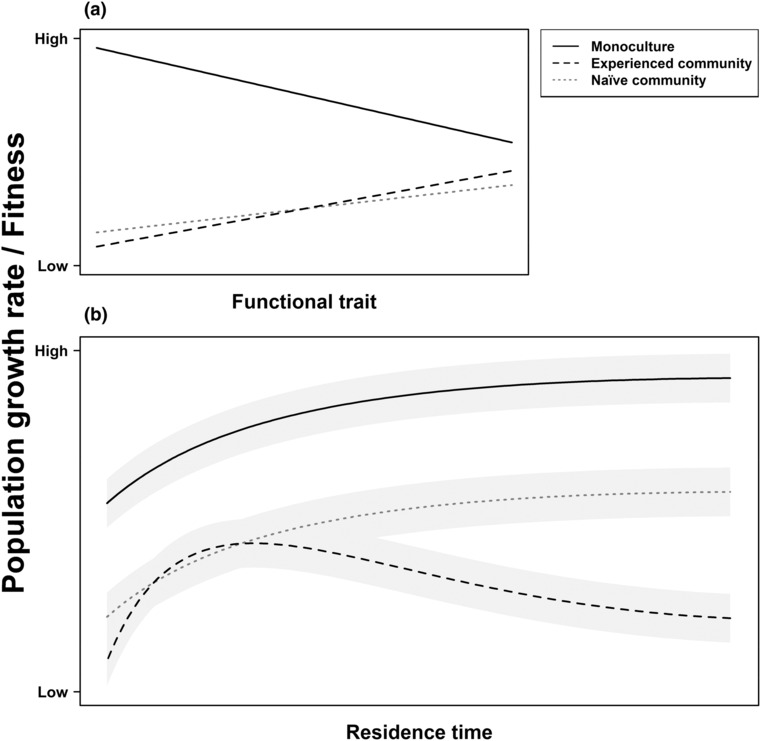
The fitness of alien plants in their new area should be determined by functional traits that are related to invasiveness and effects of competition by native communities that may depend on their eco‐evolutionary experience and thus interact with residence time of the invader. (a) Since the role of traits for fitness is likely to change depending on the habitat that is being invaded and may thus be determined by competition (e.g., low‐density monoculture vs. high‐density interspecific competition), relationships between functional traits and population growth can show a trade‐off in monoculture versus community. In this context, trait‐fitness relationships in interspecific competition should be independent of the eco‐evolutionary experience of the community and thus be similar for the experienced and naïve community. (b) As a result of intra‐ and interspecific selection to the new abiotic environment, population growth asymptotically increases with residence time in monoculture. In contrast, in a community that shares varying length of co‐existence times with the alien species (experienced community), population growth may show a unimodal response to residence time: the native community might gain eco‐evolutionary experience with the alien species and increases its competitive effects over time (i.e., builds‐up competition‐mediated biotic resistance), thereby eventually counteracting positive effects of adaptation to the new abiotic environment. In a naïve community that does not share any co‐evolutionary history with the alien species, the general negative competitive effect of the community is not expected to vary with residence time and the performance pattern of the alien species should follow that in monoculture. Note that the starting point of population growth in the new area can naturally vary before intra‐ and interspecific selection to the new abiotic environment leads to a potential fitness increase and competitive effects of the resident communities to a potential fitness decrease and may affect general levels of population growth rates (indicated by shaded areas).

In this study, we conducted a multi‐species common garden experiment with 40 Asteraceae species of varying functional traits and residence times in Germany (from recently introduced neophytes over archaeophytes to natives). We tested if the fitness of the focal species is limited by functional trade‐offs between fitness under low versus high competition intensity or by an evolutionary build‐up of competition‐mediated biotic resistance, whereby both processes are not necessarily mutually exclusive and may act simultaneously. To study these potential limits to invasion success, we measured population growth of the focal species over 2 years in a monoculture, in an experienced community (with varying potential co‐existence times between focal species and the community), and a naïve community (with a co‐existence time of zero). Thereby, monocultures present low intraspecific competition and communities present high interspecific competition (which also include intraspecific competition). According to Dietz and Edwards (2006), functional traits should cause a trade‐off between alien fitness under low versus high competition, irrespective of co‐existence time (Figure 1a). Under low competition (monocultures), intra‐ and interspecific selection imposed by the new abiotic environment plays the dominant role, whereby species of longer residence times have either changed their trait values accordingly or only those species persisted that have beneficial trait values (Brendel et al., 2021; Figure 1b). As a result, population growth of the focal species is expected to increase and eventually saturate (Figure 1b). Such local adaptation to new abiotic conditions has been shown to occur over short timescales (Colautti & Barrett, 2013). In contrast, native communities may eventually suppress population growth of invaders because of their competitive effects if they accumulate eco‐evolutionary experience. Specifically, one possible scenario is that fitness shows a unimodal response to residence time in the experienced community (Figure 1b). This is expected if alien species only become a biotic selection agent for increased biotic resistance of native plant communities after they adapted to their new abiotic environment and reached a certain level of abundance. Indeed, a build‐up of biotic resistance as a result of changing competitive interactions between the experienced community and the alien focal species encompasses highly complex reciprocal responses of co‐evolving species (Thompson et al., 2002). Importantly, if competition‐mediated biotic resistance is relevant, this unimodal effect of residence time (or any alternative patterns that suggest a limit to fitness with increasing residence time) should be detected only in competition with the experienced community, whereas the fitness‐residence time relationship for the naïve community should be parallel to that of the monoculture, given that the competitive effect of the community in this case should be independent of residence and co‐existence time (Figure 1b).

In this study, we will thus test the following hypotheses: (a) Effects of functional traits on population growth of alien plants depend on competition intensity so that traits beneficial in low‐competition monocultures are disadvantageous or unimportant under high interspecific competition. (b) Under low competition, population growth of the focal species increases with residence time, whereas in the experienced community, the fitness‐residence time relationship is unimodal due to a build‐up of competitive effects of the native community (as one aspect of biotic resistance) over time. In contrast, in the naïve community (co‐existence time of zero), the strength of competitive effects does not vary with residence time.

## 2 MATERIALS AND METHODS

### 2.1 Alien‐native species continuum

This experimental study is based on a species‐for‐time approach, for which we chose 40 annual Asteraceae species, including recently introduced neophytes, archaeophytes, and natives that arrived in Germany after the last glacial maximum (10,000–12,000 years before present; see Brendel et al., 2021; Sheppard & Brendel, 2021; Sheppard & Schurr, 2019). Neophytes represent those alien species that were introduced after the discovery of America in 1492 AD (usually rounded to 1500 AD) and archaeophytes were introduced before that date (Pyšek et al., 2004). The neophytes can be further divided into casual and established neophytes. In contrast to established neophytes, casual neophytes do not have self‐sustaining populations and rely on repeated introductions for persistence (Richardson et al., 2000). We obtained the categorization into these groups from the online database FloraWeb (Bundesamt für Naturschutz (BfN), www.floraweb.de; latest access to online database in 2016). We here do not further distinguish established and invasive neophytes, because FloraWeb does not make such a distinction as this categorization is often subjective and there is no official black list of invasive species in Germany. Note that we here consider these casual and established neophyte, archaeophyte and native species as parts of an alien‐native species continuum, rather than dividing them into invasion status categories as done in most other studies. The long and well‐documented immigration and introduction history of the Asteraceae family in Central Europe and its high proportion among established alien species in Germany (Hanspach et al., 2008), thereby allowed us to cover a wide gradient of minimum residence times (MRT) in Germany (from 32 to 12,000 years, see Figure S1 in Supporting Information). This approach enabled us to analyze temporal patterns. Furthermore, gradient designs have been shown to outperform replicated designs (such as by using categorical variables) in revealing ecological responses (Kreyling et al., 2018).

The 40 focal species are functionally similar and include all annual species occurring in ruderal and segetal habitats, which are common enough to obtain a sufficient amount of seed material (and do not originate from North America, see below). For each species, the time span between the first record in the wild and the start of the experiment in 2016 defines its MRT (sensu Rejmánek, 2000). We used the first records of each species compiled by Sheppard and Schurr (2019) from the floristic and archaeobotanical literature as well as online databases. Seeds were collected from wild populations (seeds from approx. 10 mother‐plants were sampled in 2015 and mixed before sowing) in the state of Baden‐Württemberg (the location of the common garden). Aiming to include three populations per species, we complemented the wild seed collections by seeds from botanical gardens across Germany. In total, 101 populations were included in this study (see Table S1).

### 2.2 Experimental communities

To test if the effect of competition by experimental communities on fitness/population growth of the focal species varies with functional traits and potential co‐existence time, the 40 focal species were grown in isolation as low‐density monocultures and in two plant communities. The two communities served to test if trait effects are consistent across different communities, and if the length of potential co‐existence time plays a role, by using an experienced and a naïve community. For the experienced community, we chose 12 perennial species (four grasses and eight forbs; Table S2) that belong to the grassland community association of *Festuco‐Brometea* and occur in mesic to dry calcareous grasslands in Central Europe (Ellenberg, 2009). Furthermore, they can also be found in ruderal and segetal habitats (Ellenberg, 2009) as well as on fallow land (for a case study in Germany see Klimaschewski et al., 2006), where the Asteraceae species occur, and the species are widespread across Germany (see Table S2 for species‐specific range sizes). More than 50% of the total land area in Germany is used for agriculture (Eurostat, www.ec.europa.eu; last access in 2023), whereby these segetal habitats present sources of spread of alien plants into more natural habitats such as grasslands (Kowarik, 2003). Thus, the MRT of the 40 focal species in Germany (Figure S1) serves as a measure for the length of potential co‐existence time between the Asteraceae and the experienced community species. To disentangle if competitive effects on fitness of the focal species are related to a build‐up of biotic resistance by the experienced community with MRT or to competitive abilities of the focal species that might co‐vary with MRT, we used a naïve community as control. This naïve community consists of species native to prairie grasslands of the Northeastern United States of America (Gleason & Cronquist, 1991). Particularly, they belong to the plant communities of dry to mesic prairies in the natural vegetation division “Grand Prairie Division” of the state Illinois (Mohlenbrock, 2002). The species have not been introduced to Germany (checked via Global Invasive Species Database: www.iucngisd.org; latest access to online database in 2016). The naïve community matches the experienced community at genus‐level (nine out of 12) and family‐level (the remaining three) to keep both communities as functionally similar as possible (for detailed information on species composition of the experienced community and its congeneric/confamilial counterparts of the naïve community see Table S2). However, the naïve community never interacted with the focal species. To keep the phylogenetic relatedness between each community and the focal species constant, the two communities did not include any additional Asteraceae species.

### 2.3 Experimental design

In March 2016, we set up a mesocosm‐experiment at the experimental station of the University of Hohenheim, Germany (Versuchsstation Heidfeldhof: 48°43′02.1" N, 9°11′03.1" E, 400 m a.s.l.; annual precipitation: 698 mm; mean annual temperature: 8.8°C). In this experiment, populations of each of the 40 Asteraceae focal species were exposed to three competition treatments. To investigate population growth in isolation, we established monocultures of each focal species (232 mesocosms with seeds of the focal species sown on the bare substrate to follow population growth over 2 years plus 78 mesocosms with transplanted seedlings for functional trait measurements). To investigate effects of interspecific competition on population growth, we established mesocosms of each community type (236/234 mesocosms with seeds of the focal species sown into the experienced/naïve community). Combinations of species and competition treatment were usually replicated in six mesocosms, with the number of replicates ranging from 4 to 8 (the number of replicates at population‐ and species‐level for monocultures and each community type are listed in Table S1). Mesocosms were randomly assigned to five spatial blocks and each block contained the same number of mesocosms. The distance between mesocosms within each block was 0.5 m and the distance between blocks was 1 m. Mesocosms were placed in a parcel of 180 m^2^ (60 m times 30 m) on a former meadow within a mosaic of crop fields. Before the mesocosms were arranged, the ground was covered with a weed mat to suppress growth of the surrounding vegetation. Each mesocosm consisted of a 50‐L pot (0.159 m^2^ soil surface area, 50 cm upper diameter, 38 cm lower diameter, and 40 cm height) filled with local soil (texture: 70% sand, 14% clay, and 16% silt; nutrient content: 1.81 mg/L NO^3−^, 0.015 mg/L NH^4+^, 21.36 mg/L P; pH‐value: 7.88) on top of a layer of expanding clay to improve drainage. During the growing season, the mesocosms were watered daily with an automatic drip‐irrigation system. We weeded the mesocosms before sowing and regularly throughout the experiment (once per week before and every second week after they were surrounded by an open‐top organza fabric; see below).

By the end of April, we sowed a seed‐mixture of the 12 perennial species of each community type into the respective mesocosms (at an overall density of 3 g/m^2^). The seeds were covered with a thin layer of sand. To ensure that the total seed mass of each experienced community species was comparable to its naïve counterpart, we determined the number of sown seeds per species based on the species' per‐seed mass (for seed mixtures see Table S2 and for further compositional characteristics of each community type see Appendix S1 and Figure S2). In late June, when the communities were fairly well established, we added 20 seeds from a given Asteraceae population to each community and monoculture mesocosm (i.e., only one population of one Asteraceae species was introduced to a mesocosm) to initiate population growth of the focal species at the same time across all three competition treatments. Unlike for the community mesocosms, where the seeds were sown into already established communities, we did not establish monocultures prior to sowing the seeds into the monoculture mesocosms. Hence, the initial population size of the focal species *S*_0_ equals 20 seeds across all three competition treatments. Given this small initial population size and the large size of the mesocosms (50‐L pots), we consider effects of intraspecific competition in monocultures to be at least initially low. Before the first seeds of the study species ripened in 2016, we surrounded each mesocosm by an open‐top organza fabric (see Figure S3, also for the aforementioned spatial arrangement of mesocosms) to prevent seed immigration and emigration (after population growth was initiated in 2016, we did not add any additional seeds of the Asteraceae focal species or community species), without excluding light and pollinators.

### 2.4 Measures of demography and population dynamics

Population growth was quantified as the change in seed number per mesocosm over time. As annuals, our focal species do not reproduce vegetatively (Hirose et al., 2005; plus personal observation). Thus, to follow the dynamics of each experimental Asteraceae population over 2 years, we estimated the seed number per mesocosm at the end of each year (*S*_1_ and *S*_2_, respectively) as the product of total capitula number in late October and average seed number per capitulum (from Brendel et al., 2021). Annual growth rates of the seed populations (finite rate of increase) were quantified as *λ*_*t*_ = *S*_*t*+1_/*S*_*t*_, according to Venable and Brown (1988). Given that the density of the initial population was low (*S*_0_ = 20 seeds per mesocosm), population growth rate in the first year, *λ*_0_ = *S*_1_/*S*_0_ represents an estimation of the intrinsic (density‐independent) rate of increase in monoculture mesocosms. Together with dispersal distance, *λ*_0_ determines the spread rate (Skellam, 1951) and is thus a key driver of invasion success. Since the focal species are annual, *λ*_0_ includes two demographic components, the transition from seed to plant (establishment) and the transition from plant to seed (fecundity; Brendel et al., 2021). Consequently, the number of established focal individuals per mesocosm at the end of the first growing season (*N*_1_) was used to break down *λ*_0_ into establishment (*E*_0_ = *N*_1_/*S*_0_) and fecundity (*F*_0_ = *S*_1_/*N*_1_). For each mesocosm, we thus calculated *λ*_0_, *λ*_1_, *E*_0_, and *F*_0_ as measures of population dynamics and demographic performance. We did not calculate *λ*_0_ for the few cases when a focal species did not produce any mature seeds in the first growing season in any mesocosm across all populations (and thus did not complete their life cycle in any mesocosm; this reduced the sample size from initially 40 species and 101 populations to 36 species and 94 populations, see Table 1). In this way, we avoided that *λ*_0_ = 0 was assigned to species whose seed‐set was restricted by the relatively short growing season in the first year (lasting from end of June to end of October due to logistical challenges beyond our control that delayed the experimental set‐up; Figure S4) or because they are facultative annuals (Brendel et al., 2021). If only some mesocosms of a given focal species did not produce a seed‐set, however, *λ*_0_ = 0 was retained as in this case a population growth rate and fecundity of zero is likely a response to the competitive effects of the interacting community. For the analyses of *E*_0_, we used all 40 species and 101 populations. Note that while we present data on *λ*_1_ in the Supplementary Information, we are careful not to over‐interpret these findings. Given the limited size of the mesocosms, *λ*_1_ strongly depended on population size after the first year (and thus on *λ*_0_), obscuring effects of traits and biotic resistance.

**TABLE 1:** Properties of the models relating finite rate of increase (*λ*_0_) to three functional traits (linear and quadratic effect of log‐transformed seed mass, maximum height and specific leaf area), competition treatment, and their interaction (top row); and to minimum residence time (MRT), competition treatment (contrasting monoculture vs. experienced/naïve community), and their interaction (bottom row).

Model	Competition treatment	Mean *R* ^2^ (95% credible interval)		Mean phylogenetic signal (95% credible interval)	Sample size (species, populations, mesocosms)
		Marginal	Conditional	Pagel's lambda	
Functional traits	Monoculture versus experienced community	0.68 (0.61, 0.74)	0.81 (0.78, 0.84)	0.25 (0.002, 0.46)	36, 94, 436
	Monoculture versus naïve community	0.66 (0.59, 0.72)	0.82 (0.78, 0.85)	0.26 (0.002, 0.48)	36, 94, 434
Minimum residence time	Monoculture versus experienced community	0.62 (0.54, 0.69)	0.76 (0.73, 0.81)	0.23 (0.001, 0.49)	36, 94, 436
	Monoculture versus naïve community	0.60 (0.52, 0.67)	0.77 (0.72, 0.82)	0.22 (0.001, 0.51)	36, 94, 434

*Note*: Corresponding analyses for other demographic performance measures are given in Supporting Information Table S3 (functional traits) and Table S4 (MRT).

### 2.5 Functional trait measurements

We measured seed mass, maximum height, and specific leaf area (SLA) as three major axes of ecological strategies in plants (Westoby, 1998). For an extended set of 46 Asteraceae species (including the 40 focal species plus 6 North American neophytes), low seed mass and intermediate height maximized population growth and fecundity in monoculture mesocosms (Brendel et al., 2021). A high seed mass increases seedling establishment in temperate grasslands (Moles & Westoby, 2004), but usually trades off with reproductive output (Moles et al., 2004; see Figure S5 for the trade‐off between seed mass and seed number in our focal species). An investment in height leads to a greater light interception (Falster & Westoby, 2003), and low SLA is related to a more efficient resource acquisition (Westoby, 1998). Thus, we expect these three functional traits to also be relevant for population growth in interspecific competition, albeit with different optimal trait values (see Figure 1a‐c).

For all trait measurements (see also Brendel et al., 2021), we followed the standard protocols of Pérez‐Harguindeguy et al. (2013). Before starting the experiment, we determined average seed mass at population‐level (based on six times 20 seeds using a high precision balance, accuracy of 10^−4^ g). For population‐level measurements of maximum height and SLA, additional monoculture mesocosms with transplanted seedlings were established (Brendel et al., 2021). In late June 2016, we transplanted six seedlings (previously grown in the same soil as used for the mesocosms in germination trays for 6 weeks in greenhouses next to the common garden facility) of each study species into two empty mesocosms. Whenever feasible, we evenly assigned the populations to the six individual plants (i.e., three populations leading to two individuals each per mesocosm). At the end of October 2016, we measured the height of 463 transplanted individuals (that survived from initially 466 individuals). During August 2016, we collected two leaves from each individual with at least four fully developed leaves (445 individuals). All leaves were scanned and their area was measured using ImageJ2 (Rueden et al., 2017). Afterward, the leaves were dried (at 70°C for 72 h) and weighed to calculate SLA (mm^2^/mg) at population‐level. Due to low germination rates, we could only measure five individuals for *Cyanus segetum* per mesocosm and did not have any transplanted individuals to measure for *Crepis tectorum*. For the latter species, we thus used the individuals developed from seeds. We sampled two leaves in three random mesocosms and measured the tallest individual in each mesocosm. For four populations (of four species) used to assess demographic performance, no matching transplants were available. We thus used the corresponding species‐level average of SLA and maximum height. The trait data are available from the TRY plant database (Kattge et al., 2020).

Note that a reviewer of a previous version of this manuscript suggested to also measure traits in competition treatments. While we appreciate that traits may change depending on the environment in which a species grows, we did not do this, because using traits measured on the individuals for which performance is assessed leads to a certain circularity between explanatory and response variable. Moreover, Ferenc and Sheppard (2020) found that individual‐level traits (i.e., traits measured on each individual in a specific competition context) generally did not explain variation in pairwise alien plant interactions better than species‐level traits (i.e., traits measured on separate single individuals, averaged to one value per species).

### 2.6 Statistical analyses

Data analyses were performed in R 3.5.1 (R Core Team, 2018). We used phylogenetic generalized linear mixed models (GLMMs) to analyze the two measures of population growth (*λ*_0_ and *λ*_1_) as well as individual demographic rates in the first year (*E*_0_ and *F*_0_). To directly quantify competition‐mediated biotic resistance, one has to compare a treatment with inter‐ and intraspecific competition to a control of only intraspecific competition's own species (i.e., monocultures) and initiate population growth at a constant number of seeds across all treatments. Hence, we quantified competition‐mediated biotic resistance (as well as relationships between functional traits and population dynamics) via two separate analyses that contrasted the experienced and naïve communities, respectively, to the control. However, as suggested by a reviewer of an earlier version of this manuscript, we also performed analyses with a single model including one variable of competition treatment with three levels (i.e., monoculture vs. experienced community vs. naïve community) and tested if the relationship between functional traits and *λ*_0_ of the focal species depends on the type of competition (Table S6) or the effect of competition‐mediated biotic resistance depends on residence time (Table S7). These analyses did not qualitatively change the results and will thus not be further reported in the manuscript.

To test our first hypothesis that the relationship between functional traits and performance of the focal species depends on the type of competition (Figure 1a), we entered competition treatment (monoculture vs. experienced community), the linear and quadratic term of seed mass, maximum height, and SLA as well as the interaction between competition treatment and each functional trait as fixed‐effects into the GLMMs. We ran these GLMMs for each measure of population growth and demographic rate. To further investigate if the relationship between functional traits and performance of the focal species follows the same pattern irrespective of the community type, we repeated the analyses using data from monocultures and naïve communities. In all GLMMs, functional traits were log‐transformed, scaled, and centered. To ensure our results are robust, we performed control analyses for finite rate of increase (*λ*_0_) only including wild populations since the seeds obtained from botanical gardens were not grown under entirely natural conditions.

To test our second hypothesis that the effect of competition with the community on performance of the focal species varies with MRT (Figure 1b), the GLMMs included competition treatment (monoculture vs. experienced community), the linear and quadratic term of MRT, and the interaction between competition treatment and each MRT‐term as fixed‐effects. We ran these GLMMs for each measure of population growth and demographic rate. To distinguish between potential effects based on length of co‐existence time versus competitive abilities co‐varying with MRT, we furthermore conducted control analyses comparing the monoculture to the naïve community. In all GLMMs, MRT was log‐transformed, scaled, and centered. We also performed control analyses for *λ*_0_ only including wild populations. As our analyses compare many different species (albeit of the same family, life form, and habitat), some of which are more closely related than others, we accounted for the phylogenetic relatedness among the focal species in our models. We used Pagel's lambda correlation structure (Pagel, 1999) in Bayesian GLMMs fitted with Markov chain Monte Carlo methods (MCMCglmm package; Hadfield, 2010). We extracted information on phylogenetic relatedness from the Daphne Phylogeny (Durka & Michalski, 2012) by means of the R‐packages picante (Kembel et al., 2010) and phytools (Revell, 2012). All GLMMs furthermore included experimental block and population nested in species as random effects.

To analyze *λ*_0_, *λ*_1_, and *F*_0_, we used Gaussian GLMMs with non‐informative priors for the variance components of each random effect (corresponding to an inverse‐Gamma distribution with shape and scale parameters equal to 0.01). To meet the model assumptions on residuals, we log(*x* + 1)‐transformed *λ*_0_, *λ*_1_, and *F*_0_. For the analyses of *E*_0_, we performed binomial GLMMs to contrast establishment success (*N*_1_) and failure (*S*_0_ ‐ *N*_1_). For the variance of each random effect, we used an inverse‐Wishart prior (with shape and scale parameters equal to 0.001). All GLMMs ran for 1,000,000 iterations with a burn‐in phase of 250,000 and a thinning interval of 500 (MCMC consistently converged). For fixed effects, we followed the default settings (Hadfield, 2010) and used a normal prior with a mean of zero and a very large variance (10^10^). We considered a model term to be significant, if its 95% credible interval (CI) did not overlap zero.

## 3 RESULTS

### 3.1 Interspecific competition modifies the effect of functional traits on fitness

**FIGURE 2:**
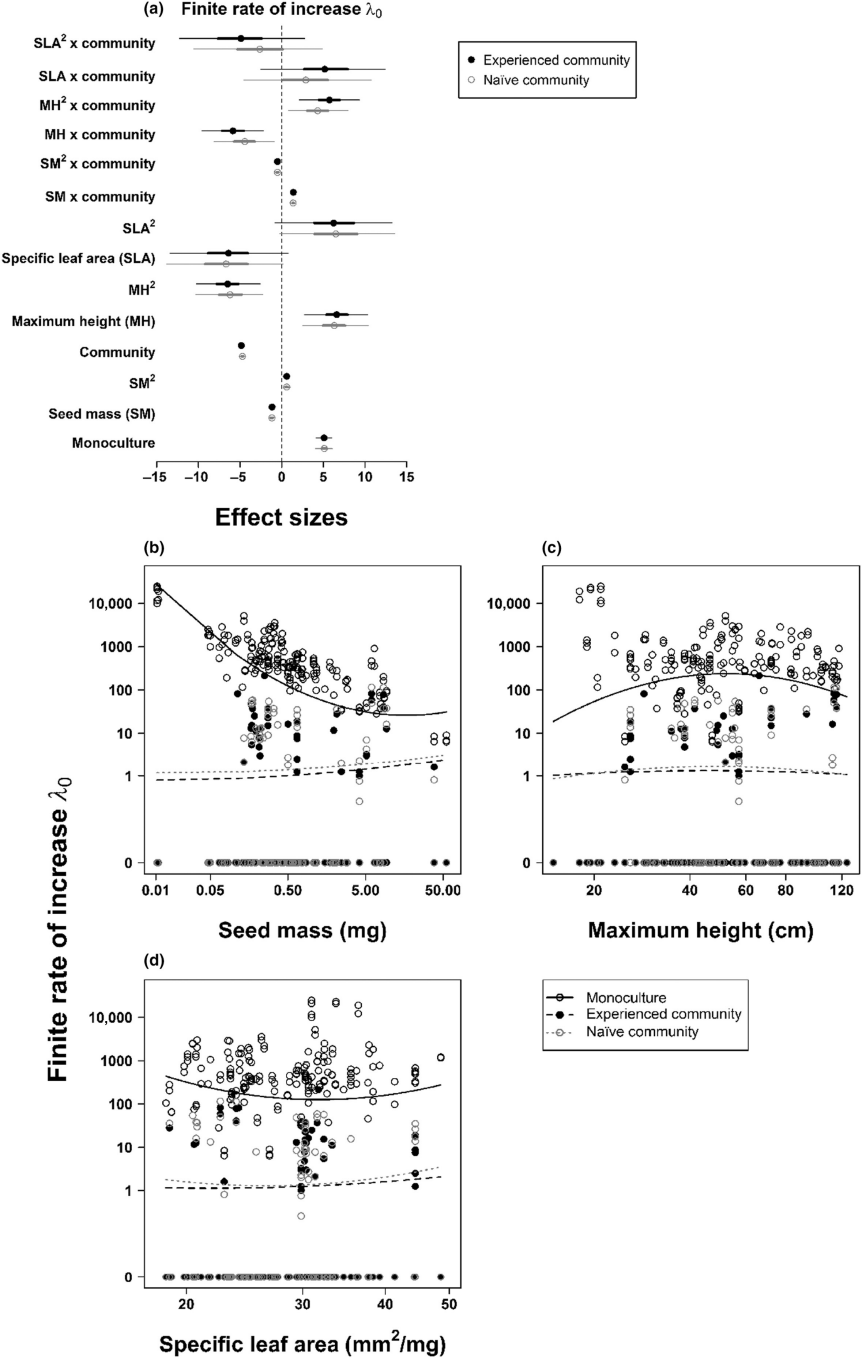
Effects of functional traits (linear and quadratic term of seed mass, maximum height, and specific leaf area), competition treatment (contrasting monoculture vs. experienced/naïve community, whereby monoculture represents the intercept, i.e., reference level, of the model), and their interaction on the finite rate of increase (*λ*_0_). (a) Effect sizes of the model contrasting monoculture versus experienced community are shown in black and effect sizes of the model contrasting monoculture versus naïve community are shown in grey. Note that the monoculture effect sizes refer to the intercept of the respective model and the community effect sizes refer to the contrast of monoculture versus community. Circles show the posterior mean effects. Thick lines represent the 68% inner credible intervals and thin lines the 95% outer credible intervals. We consider effects to be significant, if the 95% outer credible intervals do not overlap zero. (b–d) Relationships between functional traits and the finite rate of increase (*λ*_0_) in monoculture and the two community types (experienced and naïve community). Predictions of the model contrasting monoculture versus experienced community are shown in black (solid line: monoculture; dashed line: experienced community). Predictions for the naïve community (based on the model contrasting monoculture vs. naïve community) are shown as grey dotted line. Both models have identical effect sizes for monoculture, thus only one prediction is shown. Predictions are based on the full model with the other explanatory variables set to their mean value (i.e., zero, since the functional traits were scaled and centered, allowing the response of *λ*_0_ to any given trait to be interpreted independently of the other trait variables in the respective model). Note that only interactions between functional traits and competition treatment (monoculture vs. community) in (b) and (c) are significant. All axes are shown on log‐scale.

The relationship between seed mass and the finite rate of increase (*λ*_0_) differed strongly between monocultures and experienced communities: *λ*_0_ strongly decreased with seed mass in monoculture and slightly increased with seed mass in the experienced community (Figure 2a,b). In monoculture, *λ*_0_ was predicted to be maximal for the lowest seed mass measured whereas in the experienced community, *λ*_0_ was optimal for the highest seed mass measured (Figure 2b). Very similar results were obtained when comparing monocultures and naïve communities (Figure 2a,b). The contrasting effects of seed mass on *λ*_0_ in the presence and absence of interspecific competition match our hypothesis of divergent selection on functional traits in low‐ versus high‐competition environments.

The relationship between maximum height and *λ*_0_ also strongly differed between monocultures and interspecific competition (Figure 2a). While maximum height had a clear unimodal effect on *λ*_0_ in monocultures, this effect disappeared in competition with both naïve and experienced communities (Figure 2c). In contrast, we did not detect clear effects of specific leaf area on *λ*_0_ (Figure 2a,d). The functional trait models explained a high proportion of variance in (log‐transformed) *λ*_0_ (Table 1).

The response of *λ*_0_ to seed mass and maximum height was mostly driven by variation in fecundity (*F*_0_). For *F*_0_, the relationships with seed mass and maximum height differed between monoculture and interspecific competition in a similar manner as for *λ*_0_ (Figures S6 and S7). In contrast, establishment (*E*_0_) showed different and weaker responses to functional traits and competition treatments (Figures S6 and S7). Finally, we did not detect any clear effects of functional traits on population growth rate in the second year (*λ*_1_; Figures S6 and S7). The control analyses for *λ*_0_ only including wild populations did not qualitatively change the results (Table S5 and Figure S10), and neither did the single analysis across all competition treatments (Table S6).

### 3.2 Competitive effects of native communities do not vary with residence time of the invaders

**FIGURE 3:**
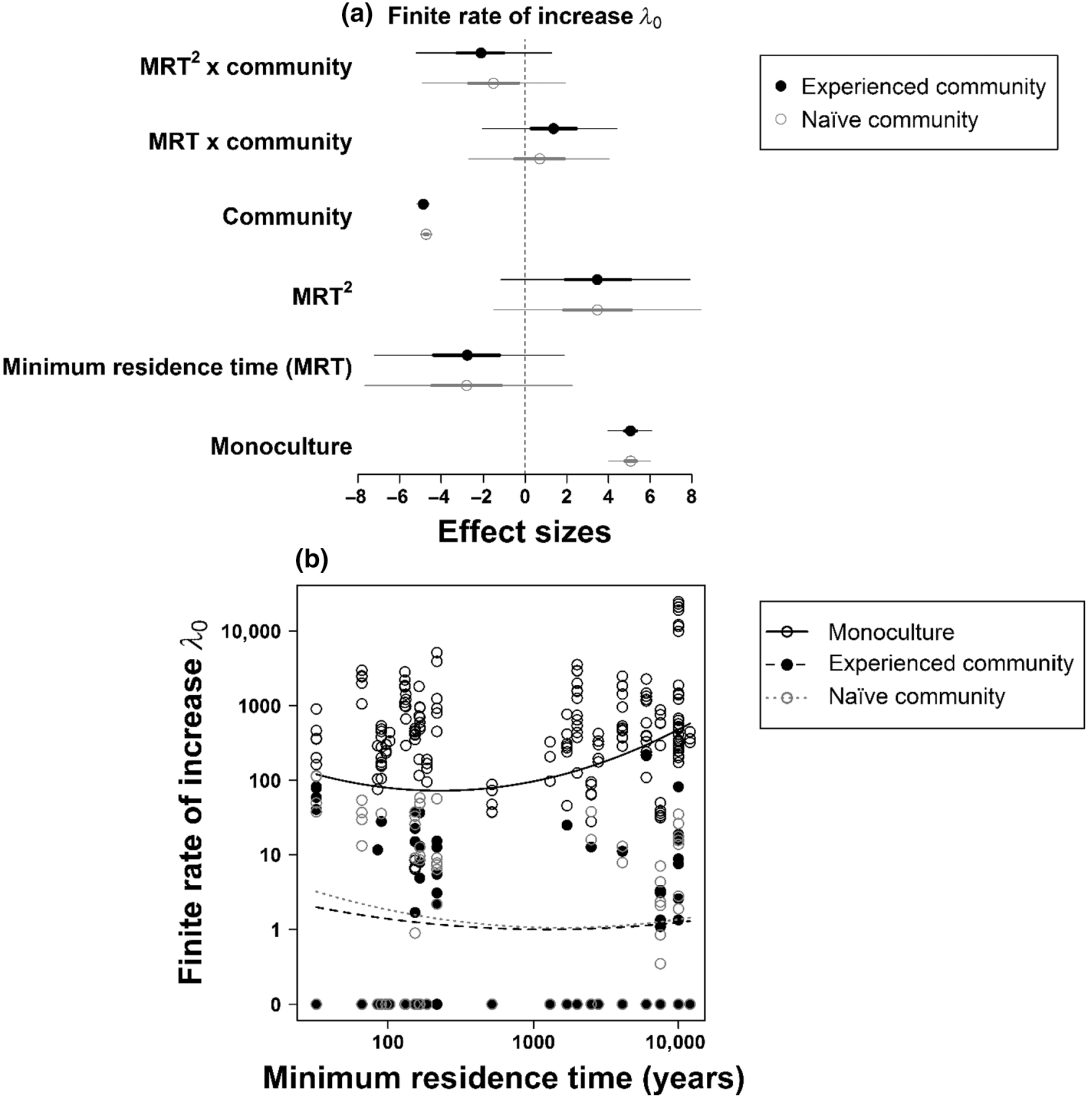
Effects of minimum residence time (MRT, linear and quadratic term), competition treatment (monoculture vs. experienced/naïve community, whereby monoculture represents the intercept, i.e., reference level, of the model), and their interaction on the finite rate of increase (*λ*_0_). (a) Effect sizes of the model contrasting monoculture versus experienced community are shown in black and effect sizes of the model contrasting monoculture versus naïve community are shown in grey. Note that monoculture effect sizes refer to the intercept of the respective model and the community effect sizes refer to the contrast of monoculture versus community. Circles show the posterior mean effects. Thick lines represent the 68% inner credible intervals and thin lines the 95% outer credible intervals. We consider effects to be significant, if the 95% outer credible intervals do not overlap zero. (b) Predictions of the model contrasting monoculture versus experienced community are shown in black (solid line: monoculture; dashed line: experienced community). The prediction of competition by the naïve community (based on the model contrasting monoculture vs. naïve community) is shown as dotted grey line. Both models have identical effect sizes for monoculture, thus only one prediction is shown. All axes are shown on log‐scale.

Competition by both the experienced and naïve community strongly reduced the finite rate of increase (*λ*_0_) of the focal species (Figure 3). Establishment (*E*_0_), fecundity (*F*_0_), and population growth in the second year (*λ*_1_) were also significantly lower in both community types than in the monoculture (Figures S8 and S9). The strong competitive effects of both community types may be explained by most community species reaching high abundances (Appendix S1 and Figure S2). Both community types reached high total cover (Figure S2), which slightly differed between the experienced and naïve community in the first year (mean ± standard deviation; experienced: 86% ± 10%, naïve: 73% ± 11%) but became very similar in the second year (experienced: 99% ± 2%, naïve: 94% ± 6%).

Under interspecific competition with the experienced community, *λ*_0_ did not show a unimodal response to minimum residence time (MRT; quadratic MRT‐interaction‐effect: posterior mean = −2.12, 95% credible interval = −5.10–1.35; Figure 3a). Instead, we found a similar response of *λ*_0_ to MRT in both community types: in the experienced and the naïve community, the focal species with longest MRTs in Germany tended to have the lowest *λ*_0_ (Figure 3b). In contrast, in monoculture, *λ*_0_ increased with MRT (Figure 3b). This contradicts our hypothesis of a build‐up of biotic resistance by the native (experienced) community over time. The respective models explain a high proportion of variance in (log‐transformed) *λ*_0_ (Table 1). We also did not find significant interactions between MRT and competition treatment for the other demographic performance measures *E*_0_, *F*_0_, and *λ*_1_ (Figures S8 and S9). Note that in monoculture, the slight decrease at very low residence times before *λ*_0_ increases (Figure 3b) could be due to variation in the starting point of population growth (as shown in Figure 1b) caused by casual neophytes that usually do not have stable populations. However, we note that to test this would require to systematically analyze differences in population growth between casual and established neophytes of the same residence time, which is not part of our study. In general, the effects of all explanatory variables on all performance measures were estimated to be similar when comparing monocultures to either experienced or naïve communities. The control analyses for *λ*_0_ only including wild populations did not qualitatively change the results (see Table S5 and Figure S11). The interaction between competition treatment and MRT (Figure S11a) reveals a similar decrease in *λ*_0_ with MRT in both the experienced and naïve community (Figure S11b). This further supports our finding that competitive effects of the communities do not vary with residence time of the focal species. Furthermore, the single analysis of the competitive effects of the communities on *λ*_0_ across all competition treatments did also not qualitatively change the results (Table S7).

## 4 DISCUSSION

By experimentally comparing the population dynamics of 40 alien and native plant species in monocultures and in either experienced or naïve plant communities, we found strong reductions in finite population growth of our focal species under interspecific competition. In line with our first hypothesis, interspecific competition by the communities markedly altered trait effects on population growth (Figure 2 and Figure S7). However, regarding our second hypothesis, we did not find evidence for a potential build‐up of competition‐mediated biotic resistance over time. Experienced communities did not exert greater competitive effects if they shared a longer potential co‐existence time with the focal species (Figure 3). Moreover, experienced and naïve communities had very similar effects on fitness and population growth, as well as trait‐fitness relationships, of the focal species (Figure 3 and Figure S8).

In the following, we discuss the potential causes and consequences of these findings.

### 4.1 Interspecific competition alters trait‐fitness relationships in alien plants

In line with our first hypothesis, we found significant differences in the relationships between functional traits and fitness of the focal species between monocultures and communities. Specifically, in monoculture, finite population growth and fecundity decreased with seed mass. The latter is expected given the trade‐off between seed mass and seed number (Moles et al., 2004; Figure S5). For the same set of Asteraceae species (plus 6 additional neophytes originating from North America), Brendel et al. (2021) previously detected a strongly negative relationship between seed mass and population growth in monocultures. We confirm this relationship for our slightly smaller species set, although it levels off at high seed mass values (Figure 2b). In contrast, under high interspecific competition, regardless of the community type, large‐seeded focal species showed highest values of finite population growth and fecundity (Figure 2b and Figure S7d, respectively). This matches the hypothesis of Dietz and Edwards (2006), who postulated that during the invasion process, alien plants experience divergent selection in low‐ versus high‐competition environments. Trait values enabling high fecundity and fast spread (e.g., low seed mass) are advantageous in low‐competition ruderal habitats but become disadvantageous under high interspecific competition.

Indeed, we found such a seed‐mass‐mediated trade‐off between population growth in low‐ versus high‐competition habitats. As expected from intra‐ and interspecific selection for ruderality in low competition, Brendel et al. (2021) showed that seed mass of the Asteraceae species converged with increasing residence time toward values that maximized population growth (*λ*_0_) and consequently, *λ*_0_ increased with their residence time. We also show this advantage of low seed mass in conditions of low competition. However, under high interspecific competition (additionally to low intraspecific competition) with the communities, low seed mass instead leads to fitness reductions (Figure 2b and Figure S7d), with many of our focal species (being mostly ruderal, annual species) not persisting over 2 years in the experimental communities. This might be because annuals over longer‐term are expected to be outcompeted by perennial species. Indeed, we did not find a trait‐mediated trade‐off for population growth rate in the second year (*λ*_1_; see Figure S7). Given the limited size of the mesocosms, *λ*_1_ strongly depended on population size after the first year and thus on *λ*_0_. Variation in population size after the first year is thus likely to obscure effects of traits and interspecific competition. Hence, the finding of *λ*_0_ more clearly suggests that divergent selection on functional traits can be imposed by interspecific interactions between species (Colautti et al., 2017) and invasion succuss might strongly depend on the ability to respond to natural selection (Lee, 2000). Thus, the expansion and impact of many alien plants may be limited because spread through low‐competition habitats (whereby disturbed sites near human settlements often being the first habitats to be colonized; McNeely, 2005) requires different traits than establishment in high‐competition habitats. This finding has important implications for management of plant invasions. It suggests that the invaders of high concern are those species that are able to escape the trait‐mediated trade‐off between performance under low and high competition and are therefore successful both at spreading rapidly in disturbed areas and at expanding into habitats of high competition, where their impact on natives is likely larger. A possible escape mechanism that allows species with high seed mass to spread rapidly in low‐competition environments may be seed dispersal by mobile animals (Nathan et al., 2008). On the other hand, small‐seeded species can increase their competitive ability if they are allelopathic or modify ecosystem properties by altering fire regimes or fixing atmospheric nitrogen.

Our results have furthermore important implications for community assembly and the co‐existence between alien and native species. In a recent study by Maron et al. (2021), small‐seeded species with high fecundity increased their abundance in low competition more than large‐seeded species with low fecundity, but showed a reduced tolerance to high interspecific competition. This seed mass‐mediated trade‐off in competitive ability, which has also been shown in our study, furthermore balanced abundances of high‐ and low‐fecundity species in a perennial grassland community and hence strongly contributed to species co‐existence (Maron et al., 2021). Moreover, Laughlin et al. (2020) recently pointed out the importance of establishing links between functional traits and population growth rates in order to advance community ecology. They call for functional community ecologists to become demographers and our study is one of the first to follow this call.

### 4.2 No evidence for a build‐up of competition‐mediated biotic resistance by experienced native communities

We expected that experienced plant communities would exert stronger competition on species with high MRT than naïve communities. However, although experienced communities developed somewhat higher cover than naïve ones (Figure S2), they did not exert stronger competition (Figure 3). In fact, both community types had surprisingly similar effects on all performance measures (Figure 3 and Figure S8). Thus, competition‐driven limits to the population growth of the studied alien plants seem to be independent of co‐evolutionary history with the native community.

This finding contradicts the expectation that over time, native communities adapt to the presence of alien species and build up biotic resistance to them (Lau, 2006; Saul et al., 2013; Saul & Jeschke, 2015; Sheppard & Schurr, 2019; Strauss et al., 2006). Our results also contradict previous empirical studies that showed higher resistance of experienced natives than naïve natives to competition with invaders (Oduor, 2013). However, most studies measured only short‐term growth differences rather than population dynamics, focused on highly abundant invasive plants (Gibson et al., 2018; Goergen et al., 2011), and did not test whether residence time (i.e., length of co‐existence time) increases biotic resistance of native species. Here, we included both common invaders (established neophytes) and less abundant aliens (casual neophytes) and covered a wide range of residence times (i.e., co‐existence times between native communities and invaders), but did not find evidence that co‐evolutionary history generally determines the strength of competition‐mediated biotic resistance of native communities. In the following, we discuss six possible explanations for these results.

Firstly, that previous studies found increased biotic resistance to invasion for experienced species whereas ours did not find such an effect might be because only highly abundant and competitive invader species rather than alien plants in general may present a large enough selective pressure to cause adaptation of native communities to new invaders. We note that to confirm this would require a systematic test of differences in competition‐mediated biotic resistance between several pairs of casual and established neophytes of the same residence time. However, in the case of our study, a separate test of casual and established neophytes as suggested by a reviewer of a previous version of this manuscript, for instance by excluding casual neophytes from the analyses, also means removing most of the lowest residence times from the alien‐native species continuum. Consequently, it would be impossible to disentangle whether differences in competition‐mediated biotic resistance are related to the absence of species with characteristics specific to casual neophytes (other than residence time) or due to missing values of lowest residence times in the alien‐native species continuum.

Secondly, an alternative explanation for our results is that such a complete lack of biotic resistance to alien species as found here may also be more common than expected, if studies that did not find an effect of increased biotic resistance with eco‐evolutionary experience are less likely published because of a publication bias. Also, some empirical studies may falsely attribute increased performance of experienced natives (or reduced performance of invaders growing with experienced natives) to a build‐up of biotic resistance due to confounding factors in observational studies or limitations of the experimental design that do not allow to conclusively demonstrate such a mechanism.

Third, in our experiment we could only test a limited set of native species in our experimental communities. Competitive response of native species in relation to eco‐evolutionary experience with alien species may, however, be native species‐specific. For instance, Mealor and Hild (2007) conducted a common garden experiment and showed that the native grass *Sporobolus airoides* consistently displayed a positive response (i.e., higher survival) to long‐term co‐existence with the invader *Acroptilon repens*, whereas the performance of the native grass *Hesperostipa comata* originating from invaded communities was not different from *H. comata* collected from non‐invaded communities. Hence, in our communities, only specific native species may have evolved competition‐mediated biotic resistance to the presence of the invaders while others did not. This might have caused the net competitive effect of the communities to be independent of co‐existence time with the invaders.

Fourth, there could also be variation in potential facilitation with native community species with length of co‐existence time, offsetting potential increases in competition. However, for a subset of our focal species, grown in mesocosms similar to the experienced community but including a legume (known for their facilitative effects due to nitrogen fixation), Ferenc et al. (2023) did not detect any facilitative effects on neophyte, archaeophyte or native Asteraceae.

Fifth, it is also likely that a build‐up of biotic resistance can more easily be detected at population level (albeit only covering considerably shorter timescales). For instance, in a pairwise competition experiment, Germain et al. (2020) showed that population growth of the invasive grass *Bromus hordeaceus* was more restricted by the native grass *Vulpia microstachys* originating from populations that have a history of co‐existence with the invader compared to non‐invaded populations.

Finally, as in our experiment, we only were able to test competitive effects of native plant species, it is possible that other components of biotic resistance such as parasitism, herbivory or plant–soil feedbacks are more important in limiting invasion success.

In a multi‐species common‐garden experiment with a smaller set of focal Asteraceae species, Sheppard and Schurr (2019) measured how survival and reproduction respond to competition by a (different) community. They found that competitive effects increased with residence time and suggested that this arises from a build‐up of biotic resistance by the native community. Furthermore, in a pairwise competition experiment, Sheppard and Brendel (2021) found that native Asteraceae tended to perform better with Asteraceae neighbors of increasing residence time (consistent with an increase in biotic resistance at the level of individual species), but only under certain soil conditions. However, our finding that naïve communities have similar competitive effects as experienced ones contradicts these findings. The weak decrease in fitness with residence time in both communities may be explained by a priori competitive ability of the focal species correlating with residence time. Accordingly, trait‐fitness relationships were also highly similar in both communities. Our study thus shows how the inclusion of a naïve community for a more robust test advances knowledge about the relevance of competition‐mediated biotic resistance.

## 5 CONCLUSIONS

We here for the first time show that seed mass has opposing effects on population growth of alien plant species under high versus low competition. This shows that the expansion and impact of invaders are limited by a seed‐mass‐mediated trade‐off between spread in low‐competition habitats versus establishment in high‐competition habitats. Invaders that are likely to escape this functional trade‐off should be of highest management concern. Furthermore, we provide a robust test of competition‐mediated biotic resistance by comparing the effect of experienced and naïve communities on population dynamics (cf. Laughlin et al., 2020) across a large set of species over 2 years. We here did not find any evidence that in our study system, an increase of competitive effects by native communities (as one aspect of biotic resistance) over time may limit population growth of alien species. Our results that expand on previous studies on interactions between alien and native species (Sheppard & Brendel, 2021; Sheppard & Schurr, 2019) thus advance both a fundamental understanding of limits to the success of alien plants and the management of alien plant invasions.

## AUTHOR CONTRIBUTIONS

**Marco R. Brendel:** Conceptualization (supporting); data curation (lead); formal analysis (lead); methodology (supporting); visualization (lead); writing – original draft (lead); writing – review and editing (equal). **Frank M. Schurr:** Conceptualization (supporting); formal analysis (supporting); methodology (supporting); supervision (supporting); writing – review and editing (equal). **Christine S. Sheppard:** Conceptualization (lead); formal analysis (supporting); funding acquisition (lead); investigation (supporting); methodology (lead); supervision (lead); writing – review and editing (equal).

## CONFLICT OF INTEREST STATEMENT

The authors declare no conflict of interest.

## Supporting information


Data S1

## Data Availability

The experimental data are given in the Figures and Supporting Information and are archived on the Dryad digital data repository (https://doi.org/10.5061/dryad.8sf7m0cvh). Data on minimum residence time were extracted from plant checklists, the floristic and archaeobotanical literature, herbaria records, and freely available online databases (Bundesamt für Naturschutz (BfN), www.floraweb.de; FlorKart, BfN, and NetPhyD Netzwerk Phytodiversität Deutschlands e.V., www.deutschlandflora.de; Kühn et al., 2004, www.biolflor.de; Naturkundemuseum Stuttgart, www.florabw.recorder‐d.de). Functional traits are available from the TRY plant trait database (Kattge et al., 2020, published in Global Change Biology).
