# Supplementary material for: Alien plant fitness is limited by functional trade‐offs rather than a long‐term increase in competitive effects of native communities
Source: Ecol Evol. 2023 Sep 1;13(9):e10468. doi: 10.1002/ece3.10468 (PMC10472529; doi:10.1002/ece3.10468)
Supplement: Supplementary file 1 — Data S1 [file ECE3-13-e10468-s001.doc]

**Supporting Information**

Alien plant fitness is limited by functional trade-offs rather than a long-term increase in competitive effects of native communities

Marco R. Brendel^1,2,*^; Frank M. Schurr^1^; Christine S. Sheppard^1^

^1^Institute of Landscape and Plant Ecology, University of Hohenheim, D-70593 Stuttgart, Germany

^2^Division of Conservation in Agriculture, German Federal Agency for Nature Conservation, D-53179 Bonn, Germany

*marco.brendel@uni-hohenheim.de

**Alien-native continuum of the Asteraceae species**

**
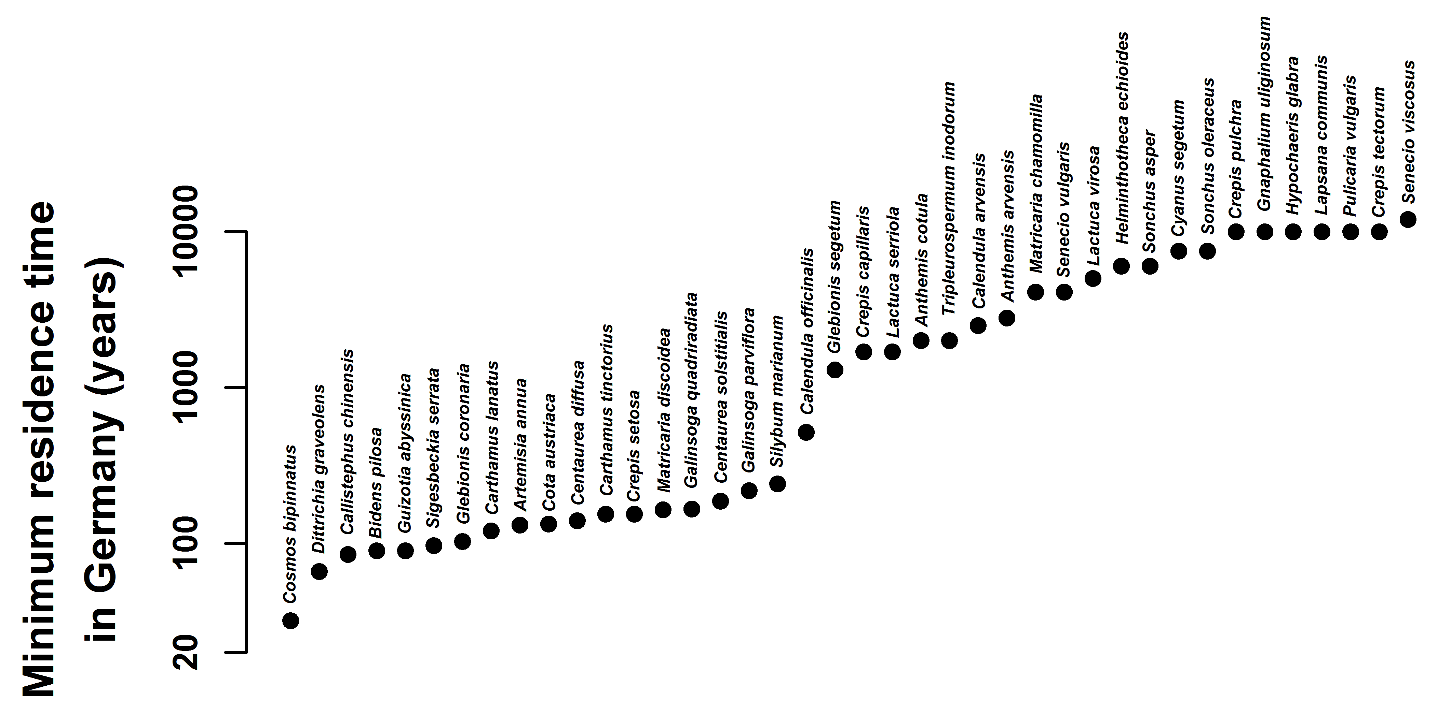
**

Figure S1: The alien-native continuum of the 40 Asteraceae species varying in their minimum residence time in Germany.

Table S1: The 40 Asteraceae species (nomenclature according to the Plant List, http://www.theplantlist.org, Kalwij 2012) and the number of mesocosms per population (seeds from wild populations and botanical gardens as indicated by city) for monoculture and each community type.

| **Asteraceae species** | **Mesocosms per population** | | |
| --- | --- | --- | --- |
|  | **Monoculture** | **Experienced community** | **Naïve community** |
| *Anthemis arvensis* L. | 2_wild_ /2_wild_ / 2* | 2_wild_ / 2_wild_ / 2* | 2_wild_ / 2_wild_ / 2* |
| *Anthemis cotula* L. | 2_Berlin-Dahlem_ / 2_Hohenheim_ / 2_Konstanz_ | 2_Berlin-Dahlem_ / 2_Hohenheim_ / 2_Konstanz_ | 2_Berlin-Dahlem_ / 2_Hohenheim_ / 2_Konstanz_ |
| *Artemisia annua* L. | 2_Bonn_ / 2_Dresden_ / 2_Konstanz_ | 2_Bonn_ / 2_Dresden_ / 2_Konstanz_ | 2_Bonn_ / 2_Dresden_ / 2_Konstanz_ |
| *Bidens pilosa* L. | 4_Dresden_ / 2_Hohenheim_ | 4_Dresden_ / 2_Hohenheim_ | 4_Dresden_ / 2_Hohenheim_ |
| *Calendula arvensis* M.Bieb. | 2_wild_ / 1_wild_ / 2_wild_ | 2_wild_ / 2_wild_ / 2_wild_ | 2_wild_ / 2_wild_ / 2_wild_ |
| *Calendula officinalis* L. | 2_wild_ / 2_wild_ / 2_wild_ | 2_wild_ / 2_wild_ / 2_wild_ | 2_wild_ / 2_wild_ / 2_wild_ |
| *Callistephus chinensis* (L.) Nees | 1_wild_ / 2_Dresden_ / 2_Tuebingen_ | 2_wild_ / 2_Dresden_ / 2_Tuebingen_ | 2_wild_ / 2_Dresden_ / 2_Tuebingen_ |
| *Carthamus lanatus* L. | 4_Dresden_ | 4_Dresden_ | 4_Dresden_ |
| *Carthamus tinctorius* L. | 2_Bonn_ / 4_Halle_ | 2_Bonn_ / 4_Halle_ | 2_Bonn_ / 4_Halle_ |
| *Centaurea diffusa* Lam. | 2_wild_ / 2_wild_ / 2_wild_ | 2_wild_ / 2_wild_ / 2_wild_ | 2_wild_ / 2_wild_ / 2_wild_ |
| *Centaurea solstitialis* L. | 2_Berlin-Dahlem_ / 4_Dresden_ | 2_Berlin-Dahlem_ / 4_Dresden_ | 2_Berlin-Dahlem_ / 4_Dresden_ |
| *Cosmos bipinnatus* Cav. | 2_wild_ / 2_wild_ / 2_wild_ | 2_wild_ / 2_wild_ / 2_wild_ | 2_wild_ / 2_wild_ / 2_wild_ |
| *Cota austriaca* (Jacq.) Sch.Bip. | 3_Hohenheim_ / 3_Potsdam_ | 3_Hohenheim_ / 3_Potsdam_ | 3_Hohenheim_ / 3_Potsdam_ |
| *Crepis capillaris* (L.) Wallr. | 2_wild_ / 2_wild_ / 2_wild_ | 2_wild_ / 2_wild_ / 2_wild_ | 2_wild_ / 2_wild_ / 2_wild_ |
| *Crepis pulchra* L. | 4_Dresden_ / 2_Konstanz_ | 4_Dresden_ / 2_Konstanz_ | 4_Dresden_ / 2_Konstanz_ |
| *Crepis setosa* Haller f. | 6_Dresden_ | 6_Dresden_ | 6_Dresden_ |
| *Crepis tectorum* L. | 6** | 6** | 6** |
| *Cyanus segetum* Hill. | 4_wild_ / 2_wild_ / 2_wild_ | 4_wild_ / 2_wild_ / 2_wild_ | 4_wild_ / 2_wild_ / 2_wild_ |
| *Dittrichia graveolens* (L.) Greuter | 2_wild_ / 2_wild_ / 2_wild_ | 2_wild_ / 2_wild_ / 2_wild_ | 3_wild_ / 2_wild_ / 2_wild_ |
| *Galinsoga parviflora* Cav. | 2_wild_ / 2_wild_ / 2_wild_ | 2_wild_ / 2_wild_ / 2_wild_ | 2_wild_ / 2_wild_ / 2_wild_ |
| *Galinsoga quadriradiata* Ruiz & Pav. | 2_wild_ / 2_wild_ / 2_wild_ | 2_wild_ / 2_wild_ / 2_wild_ | 2_wild_ / 3_wild_ / 2_wild_ |
| *Glebionis coronaria* (L.) Cass. ex Spach | 2_wild_ / 2_wild_ / 2_wild_ | 2_wild_ / 2_wild_ / 2_wild_ | 2_wild_ / 2_wild_ / 2_wild_ |
| *Glebionis segetum* (L.) Fourr. | 2_wild_ / 2_wild_ / 2_Dresden_ | 2_wild_ / 2_wild_ / 2_Dresden_ | 2_wild_ / 2_wild_ / 2_Dresden_ |
| *Gnaphalium uliginosum* L. | 4_wild_ / 2_wild_ / 2_wild_ | 4_wild_ / 2_wild_ / 2_wild_ | 4_wild_ / 2_wild_ / 2_wild_ |
| *Guizotia abyssinica* (L.f.) Cass. | 2_Bonn_ / 2_Dresden_ / 2_Hohenheim_ | 2_Bonn_ / 2_Dresden_ / 2_Hohenheim_ | 2_Bonn_ / 2_Dresden_ / 2_Hohenheim_ |
| *Helminthotheca echioides* (L.) Holub | 2_wild_ / 2_wild_ / 2_wild_ | 2_wild_ / 2_wild_ / 2_wild_ | 2_wild_ / 2_wild_ / 2_wild_ |
| *Hypochaeris glabra* L. | 6_Dresden_ | 6_Dresden_ | 6_Dresden_ |
| *Lactuca serriola* L. | 2_wild_ / 2_wild_ / 2_wild_ | 2_wild_ / 2_wild_ / 2_wild_ | 2_wild_ / 2_wild_ / 2_wild_ |

Table S1: Continued.

| **Asteraceae species** | **Mesocosms per population** | | |
| --- | --- | --- | --- |
|  | **Monoculture** | **Experienced community** | **Naïve community** |
| *Lactuca virosa* Habl. | 2_Hohenheim_ | 2_Hohenheim_ | 2_Hohenheim_ |
| *Lapsana communis* L. | 2_wild_ / 2_wild_ / 2_wild_ | 2_wild_ / 2_wild_ / 2_wild_ | 2_wild_ / 2_wild_ / 2_wild_ |
| *Matricaria chamomilla* L. | 2_wild_ / 2_wild_ / 2_wild_ | 2_wild_ / 2_wild_ / 2_wild_ | 2_wild_ / 2_wild_ / 2_wild_ |
| *Matricaria discoidea* DC. | 2_wild_ / 2_wild_ / 2_wild_ | 2_wild_ / 2_wild_ / 2_wild_ | 2_wild_ / 2_wild_ / 2_wild_ |
| *Pulicaria vulgaris* Gaertn. | 2_wild_ / 2_wild_ / 2_wild_ | 2_wild_ / 3_wild_ / 2_wild_ | 2_wild_ / 1_wild_ / 2_wild_ |
| *Senecio viscosus* L. | 2_wild_ / 2_wild_ | 2_wild_ / 2_wild_ | 2_wild_ / 2_wild_ |
| *Senecio vulgaris* L. | 2_wild_ / 2_wild_ / 2_wild_ | 2_wild_ / 2_wild_ / 2_wild_ | 2_wild_ / 2_wild_ / 2_wild_ |
| *Sigesbeckia serrata* DC. | 6_Dresden_ | 6_Dresden_ | 6_Dresden_ |
| *Silybum marianum* (L.) Gaertn. | 2_Bonn_ / 2_Rostock_ | 2_Bonn_ / 2_Rostock_ | 2_Bonn_ / 2_Rostock_ |
| *Sonchus asper* (L.) Hill | 2_wild_ / 2_wild_ / 2_wild_ | 2_wild_ / 3_wild_ / 2_wild_ | 2_wild_ / 1_wild_ / 2_wild_ |
| *Sonchus oleraceus* (L.) L. | 2_wild_ / 2_wild_ / 2_wild_ | 2_wild_ / 2_wild_ / 2_wild_ | 2_wild_ / 2_wild_ / 2_wild_ |
| *Tripleurospermum inodorum* (L.) Sch.Bip. | 3_wild_ / 2_wild_ / 2_wild_ | 2_wild_ / 2_wild_ / 2_wild_ | 2_wild_ / 2_wild_ / 2_wild_ |

* seeds received from commercial seed supplier Rieger-Hofmann GmbH (Blaufelden-Raboldshausen, Germany) in 2015

** seed source from botanical garden in Tuebingen and grown for one generation at Hohenheim
(both cases were removed for control analyses only including wild populations)

**Seed mixtures of each community type**

Table S2: Composition of the experienced and naïve community, consisting of species native to Central European grasslands and congeneric/confamilial species native to North American grasslands, respectively. Seeds originate from regional seed sources (see explanations below). The number of seeds for each species was adjusted based on the individual seed mass. In total, 477 mg seed weight per mesocosm (on a soil surface of 0.159 m^2^) refers to a sowing density of 3 g m^-2^. The low germination ability in Bromus erectus and Elymus repens (personal communication with the commercial seed supplier) explains the relatively higher amount of seeds for these species. As a measure of the range size of the experienced community species in Germany (indicated in parentheses), we calculated the proportion (%) of occupied raster-cells in Germany using the FlorKart, BfN and NetPhyD Netzwerk Phytodiversität Deutschlands e.V. database (www.floraweb.de).

| **Species composition** | | **Individual seed mass (mg)** | **Seed number per mesocosm** | **Seed weight per mesocosm (mg)** |
| --- | --- | --- | --- | --- |
| **Experienced community^1^** | **Naïve community^2^** |
| *Bromus erectus* Huds. (59.8 %) | *Bromus kalmii* A.Gray | 5.40 / 3.54 | 29 / 14 | 156.60 / 49.56 |
| *Festuca rupicola* Heuff. (15.0 %) | *Festuca paradoxa* Desv. | 0.50 / 1.23 | 20 / 20 | 10.00 / 24.60 |
| *Elymus repens* (L.) Gould (99.2 %) | *Elymus canadensis* L. | 3.00 / 5.45 | 29 / 14 | 87.00 / 76.30 |
| *Carex flacca* Schreb. (77.5 %) | *Carex bicknellii* Britton & A.Br. | 0.83 / 1.67 | 29 / 20 | 24.07 / 33.40 |
| *Galium verum* L. (71.0 %) | *Galium concinnum* Torr. & A.Gray | 0.50 / 0.47 | 50 / 50 | 25.00 / 23.50 |
| *Salvia pratensis* L. (56.5 %) | *Salvia lyrata* L. | 1.80 / 1.89 | 25 / 25 | 45.00 / 47.25 |
| *Campanula rapunculus* L. (46.0 %) | *Lobelia spicata* Lam. | 0.02 / 0.03 | 100 / 100 | 2.00 / 3.00 |
| *Potentilla argentea* L. (80.1 %) | *Potentilla arguta* Pursh | 0.10 / 0.12 | 50 / 50 | 5.00 / 6.00 |
| *Verbascum lychnitis* L. (57.1 %) | *Penstemon tubaeflorus* Nutt. | 0.10 / 0.31 | 50 / 50 | 5.00 / 15.50 |
| *Euphorbia cyparissias* L. (86.3 %) | *Euphorbia corollata* L. | 2.20 / 3.54 | 25 / 25 | 55.00 / 88.50 |
| *Silene nutans* L. (58.2 %) | *Silene regia* Sims | 0.30 / 1.23 | 50 / 25 | 15.00 / 30.75 |
| *Falcaria vulgaris* Bernh. (44.1 %) | *Thaspium trifoliatum* (L.) A.Gray | 2.00 / 3.15 | 25 / 25 | 50.00 / 78.75 |
|  |  | **Σ** | **482 / 418** | **479.67 / 477.10** |

Seed material and information on individual seed mass was obtained from commercial seed supplier ^1^Rieger-Hofmann GmbH (Blaufelden-Raboldshausen, Germany; regional seed sources “Regiosaatgut”) and ^2^Prairie Moon Nursey (Westfield, Wisconsin, USA).

The commercial seed supplier Rieger-Hoffmann provides autochthonous seed material (“Regiosaatgut”) from in total 22 regional seed sources across Germany that is mandatory for grassland restoration and in the open landscape (Durka et al., 2019).

The commercial seed supplier Prairie Moon Nursey provides seed material produced within the Midwestern prairie ecoregion that is commonly obtained for research on prairie grassland communities (Bauer, Kleczewski, Bever, Clay, & Reynolds, 2012; Larson et al., 2011).

**References**

Bauer, J. T., Kleczewski, N. M., Bever, J. D., Clay, K., & Reynolds, H. L. (2012). Nitrogen-fixing bacteria, arbuscular mycorrhizal fungi, and the productivity and structure of prairie grassland communities. *Oecologia*, *170*(4), 1089–1098. doi: 10.1007/s00442-012-2363-3

Durka, W., Bossdorf, O., Bucharova, A., Frenzel, M., Hermann, J., Hölzel, N., … Kollmann, J. (2019). Regionales Saatgut von Wiesenpflanzen: genetische Unterschiede, regionale Anpassung und Interaktion mit Insekten. Regional seed of grassland plants: genetic differences, regional adaptation and interaction with insects. *Natur und Landschaft*, *94*(4), 146–153. doi: 10.17433/4.2019.50153679.146-153

Larson, D. L., Bright, J. B., Drobney, P., Larson, J. L., Palaia, N., Rabie, P. A., … Wells, D. (2011). Effects of planting method and seed mix richness on the early stages of tallgrass prairie restoration. *Biological Conservation*, *144*(12), 3127–3139. doi: 10.1016/j.biocon.2011.10.018

**Appendix S1 Compositional characteristics of each community type**

All community species established and were abundant in the first and the second year of the experiment (only in the experienced community, *Bromus erectus* was present in the first year but absent in the second year, while *Falcaria vulgaris* was absent in the first year and present in the second year; Fig. S2a and b). In the first year of the experiment, *Salvia pratensis* and *Salvia lyrata* were the most abundant forbs in the experienced and naïve community, respectively (Fig. S2a). In the naïve community*, Elymus canadensis* was the most dominant grass (in the first and second year of the experiment), while *Festuca rupicola* and *Carex flacca* reached highest abundances among the grass species in the experienced community (Fig. S2a and b). From the first to the second year of the experiment, *Potentilla argentea* and *Potentilla arguta* increased in abundance in the experienced and naïve community, respectively (Fig. S2b). Additionally, *Penstemon tubaeflorus* showed relatively high abundances in the naïve community (Fig. S2b). The total cover of the communities, which was visually estimated, slightly differed between the experienced and naïve community in the first year (Fig. S2c) but became very similar in the second year (Fig. S2d).

**
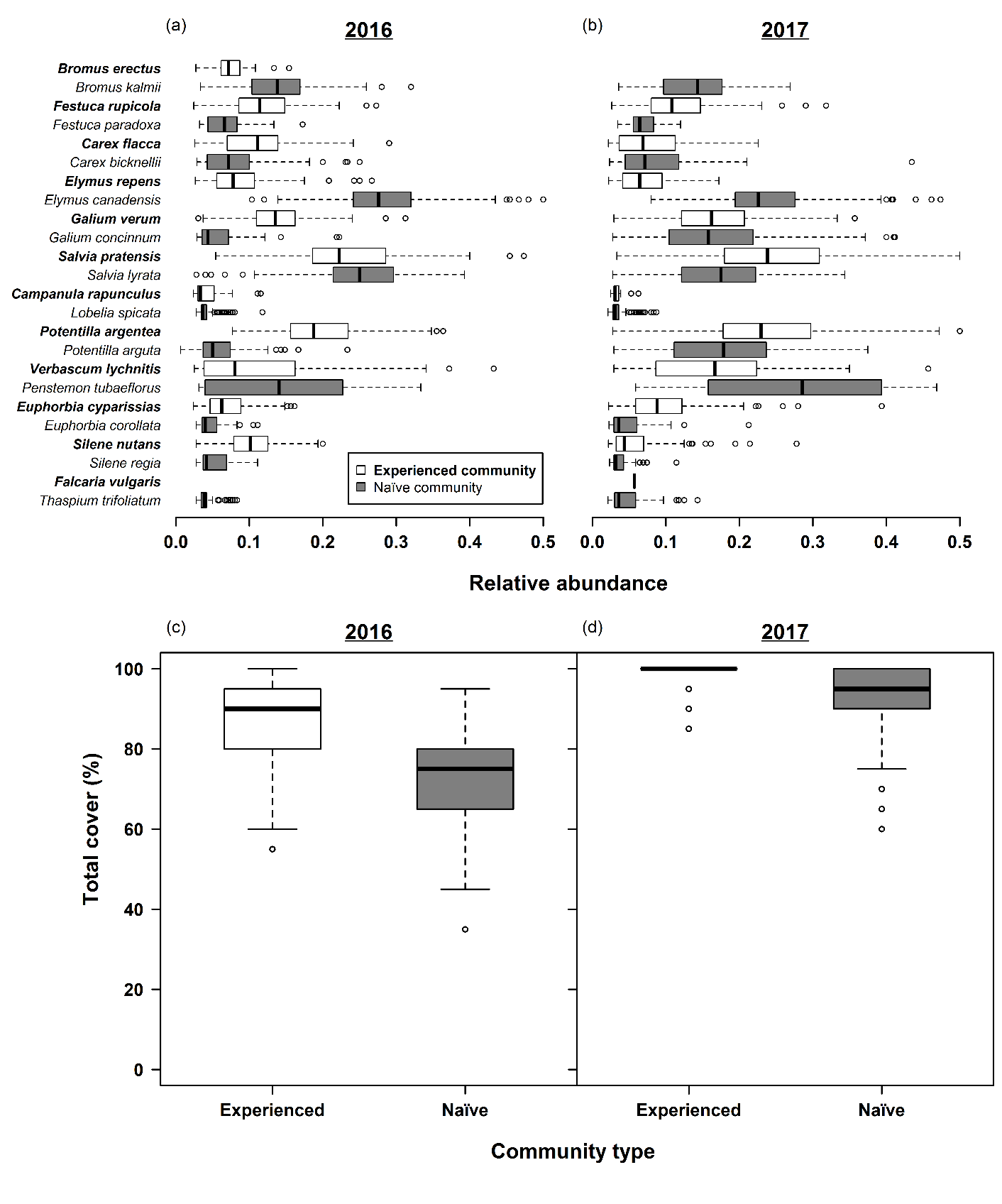
**

Figure S2: (a and b) Relative abundance*s* of community species and (c and d) total cover of each community type after the first year (2016) and second year (2017). Species of the experienced community (in a and b) are highlighted in bold. Note that *Falcaria vulgaris* was absent in the first year and *Bromus erectus* was absent in the second year. Relative abundances and total cover are based on 236/234 mesocosms for the experienced/naïve community in the first year (corresponding to the analyses of establishment *E*_0_) and 206/204 mesocosms for the experienced/naïve community in the second year (corresponding to the analyses of population growth rate in the second year λ_1_).

**Experimental design**

**
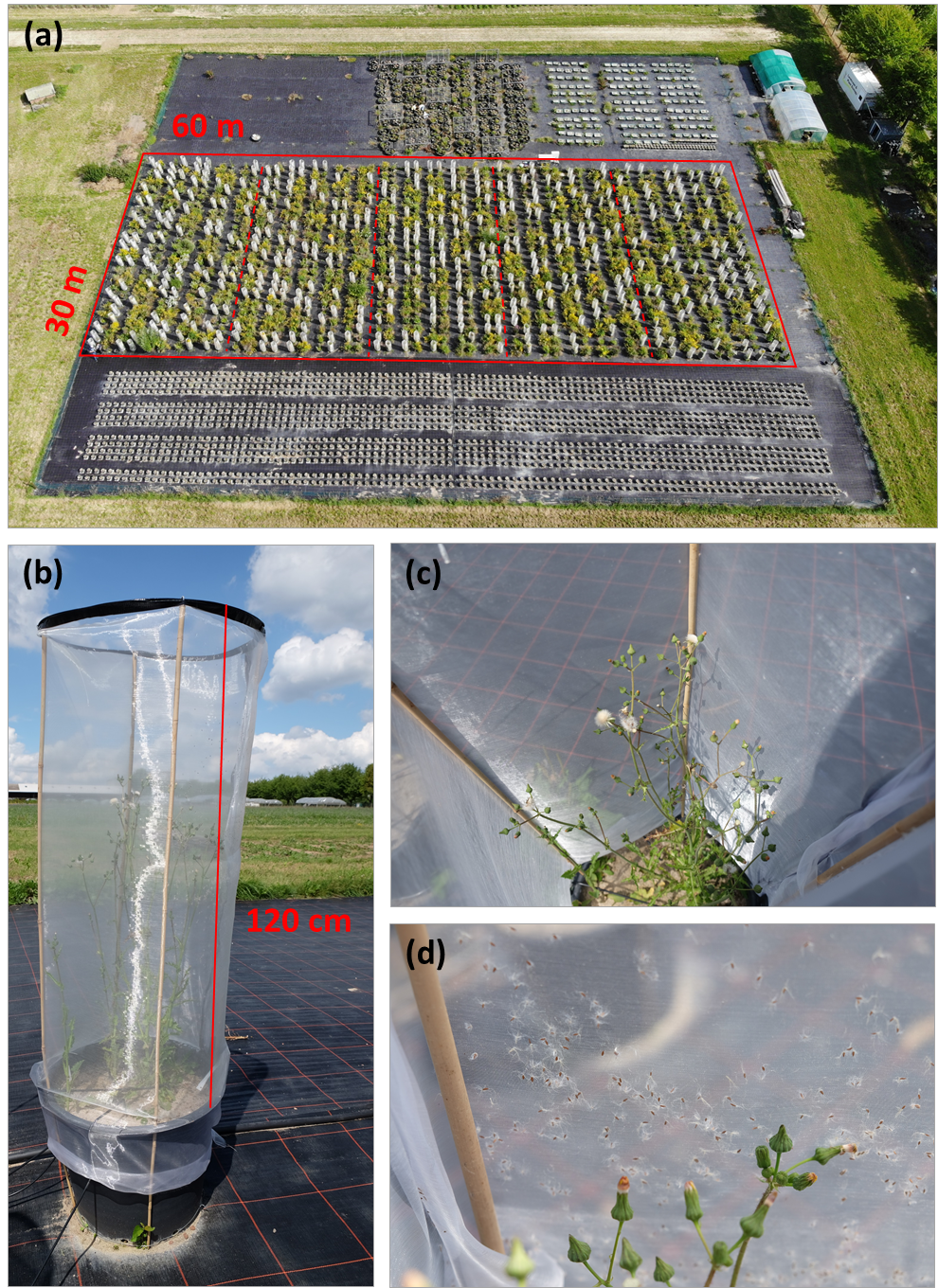
**

Figure S3: (a) Spatial arrangement of the mesocosms (random block design) used to follow population dynamics (with open-top organza fabric) and for functional trait measurements, showing the common garden experiment in the second year (2017). Note that each block also includes mesocosms used for the experiment of Brendel et al. 2021 (Global Ecology and Biogeography) and another (smaller) experiment not covered by the present study. (b) Example of an open-top organza fabric, surrounding a Sonchus asper monoculture mesocosm, which retains the developed seeds (c and d). Photo credits: (a) – ©Viktoria Ferenc; (b-d) – ©Huw Cooksley


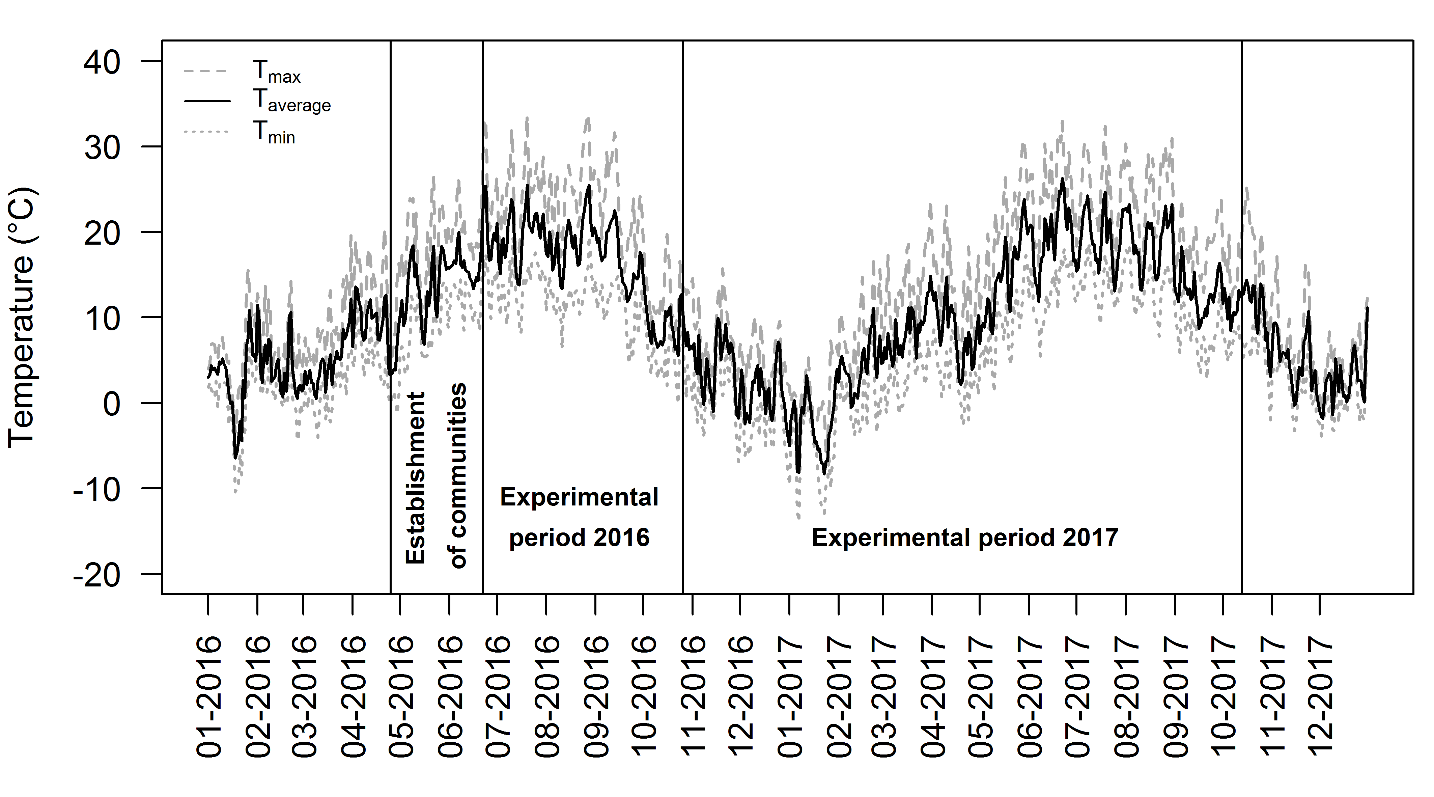


Figure S4: Maximum (T_max_), average (T_average_) and minimum (T_min_) daily temperature values (from January 1^st^, 2016, to December 31^st^, 2017, measured at 2 m above the ground) at the weather station in proximity to the field site (48° 42' 40.212'' N, 9° 11' 45.384'' E, 389 m a.s.l.), provided by the state institution on agro-meteorology (Agrarmeteorologie Baden-Württemberg, www.wetter-bw.de). The phase of community establishment (first day of sowing the seed-mixture of Central European and North American community species on April 25^th^ until first day of sowing Asteraceae seeds on June 22^nd^), the experimental period in 2016 (first day of sowing Asteraceae seeds on June 22^nd^ until last day of assessment on October 26^th^) and the experimental period in 2017 (October 27^th^, 2016, until last day of assessment on October 13^th^, 2017) are indicated.

**Trade-off between seed mass and seed number of the Asteraceae study species**

**
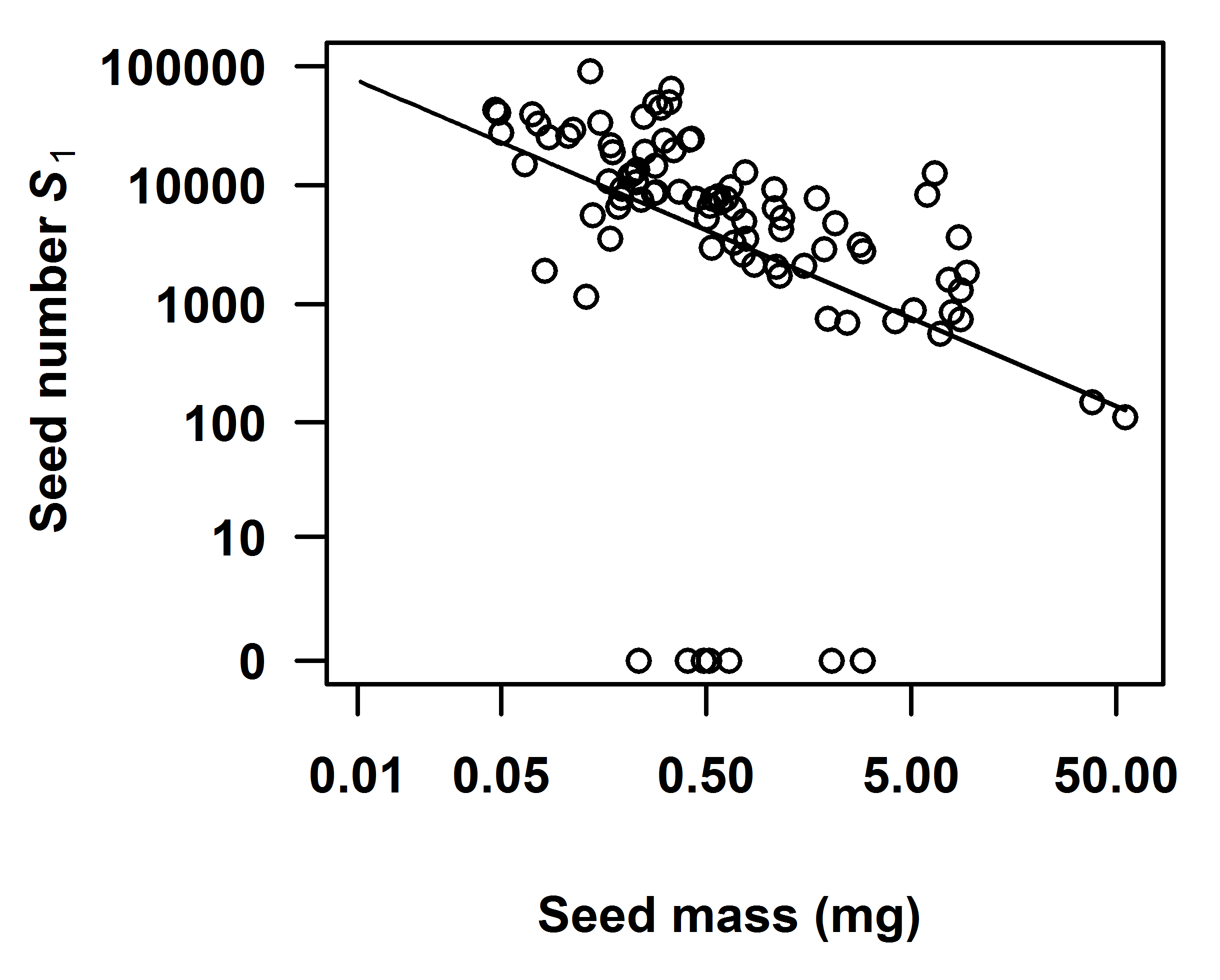
**

Figure S5: Significant effect of log-transformed seed mass on average log seed number per monoculture mesocosm at population level in the first year of the experiment.

**Effects of competition treatment on relationships between functional traits and demographic performance**

Table S3: Properties of the models relating establishment (E_0_), fecundity (F_0_) (in the first year), and population growth rate in the second year (λ_1_) to three functional traits (linear and quadratic effect of log-transformed seed mass, maximum height and specific leaf area), competition treatment (contrasting monoculture vs. experienced/naïve community), and their interaction.

|  |  | **Mean R^2^**  **(95% credible interval)** | | **Mean phylogenetic signal**  **(95% credible interval)** |  |
| --- | --- | --- | --- | --- | --- |
| **Demographic performance measure** | **Interspecific competition  with the community** | **marginal** | **conditional** | **Pagel's lambda** | **Sample size**  **(species, populations, mesocosms)** |
| Establishment *E*_0_ | monoculture  vs.  experienced community | 0.43 (0.31, 0.54) | 0.75 (0.66, 0.83) | 0.18 (0.0004, 0.48) | 40, 101, 468 |
|  | monoculture  vs.  naïve community | 0.34 (0.22, 0.47) | 0.68 (0.59, 0.78) | 0.10 (0.0003, 0.32) | 40, 101, 466 |
| Fecundity *F*_0_ | monoculture  vs.  experienced community | 0.71 (0.64, 0.76) | 0.82 (0.80, 0.86) | 0.25 (0.001, 0.46) | 36, 94, 430 |
|  | monoculture  vs.  naïve community | 0.68 (0.61, 0.74) | 0.82 (0.79, 0.86) | 0.27 (0.002, 0.49) | 36, 94, 428 |
| Population growth rate λ_1_ | monoculture  vs.  experienced community | 0.42 (0.34, 0.49) | 0.64 (0.56, 0.71) | 0.16 (0.02, 0.34) | 36, 87, 394 |
|  | monoculture  vs.  naïve community | 0.34 (0.27, 0.41) | 0.55 (0.46, 0.64) | 0.12 (0.01, 0.29) | 36, 87, 392 |

**
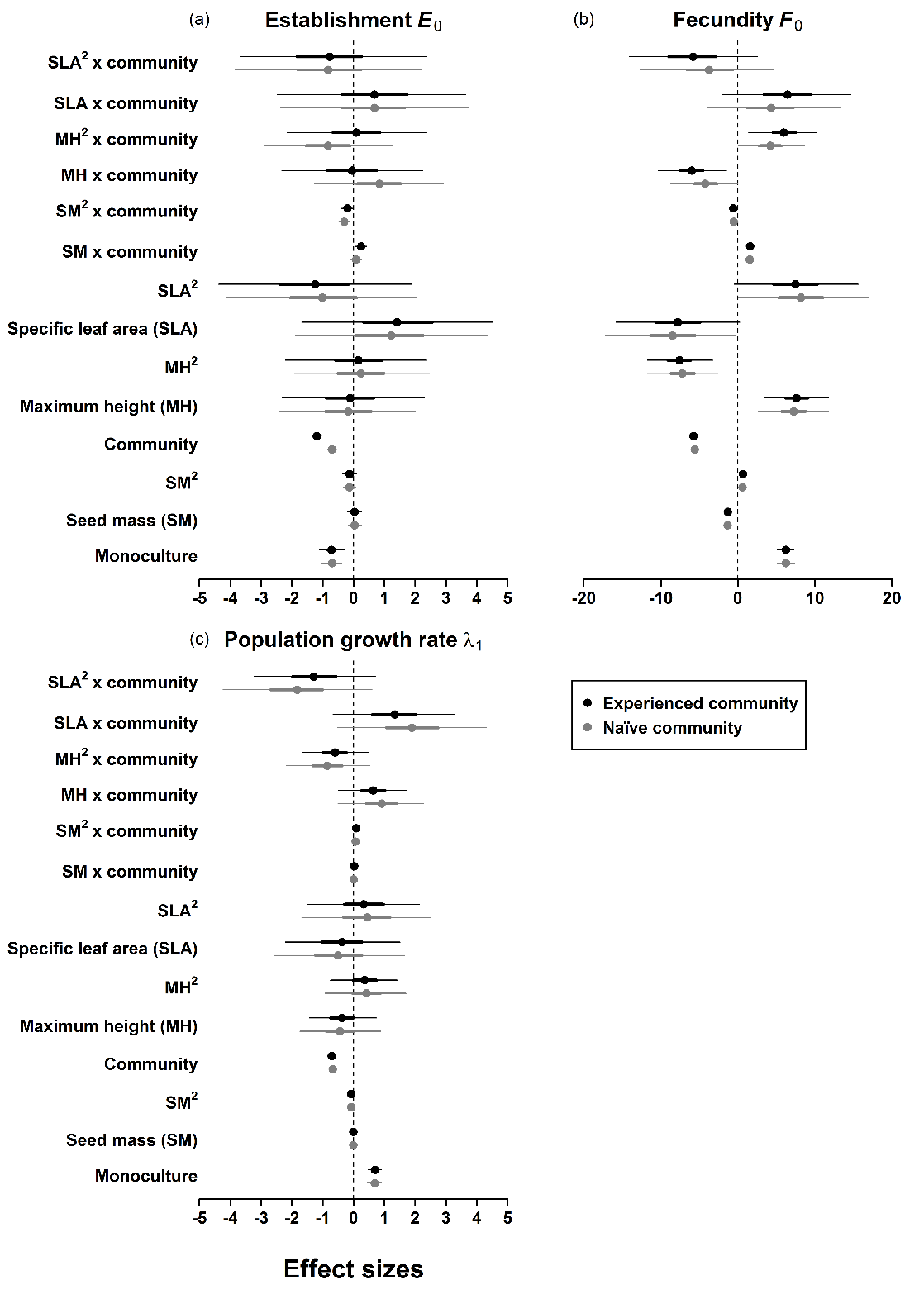
**

Figure S6: Effects of functional traits (linear and quadratic term of seed mass, maximum height, and specific leaf area), competition treatment (contrasting monoculture vs. experienced/naïve community, whereby monoculture represents the intercept, i.e. reference level, of the model), and their interaction on (a) establish­ment (E_0_), (b) fecundity (F_0_) (in the first year), and (c) population growth rate in the second year (λ_1_). Effect sizes of the models contrasting monoculture vs. experienced community are shown in black and effect sizes of the models contrasting monoculture vs. naïve community are shown in grey. Note that the monoculture effect sizes refer to the intercept of the respective models and the community effect sizes refer to the contrast of monoculture vs. community. Filled circles show the posterior mean effects. Thick lines represent the 68% inner credible intervals and thin lines the 95% outer credible intervals. We consider effects to be significant, if the 95% outer credible intervals do not overlap zero.


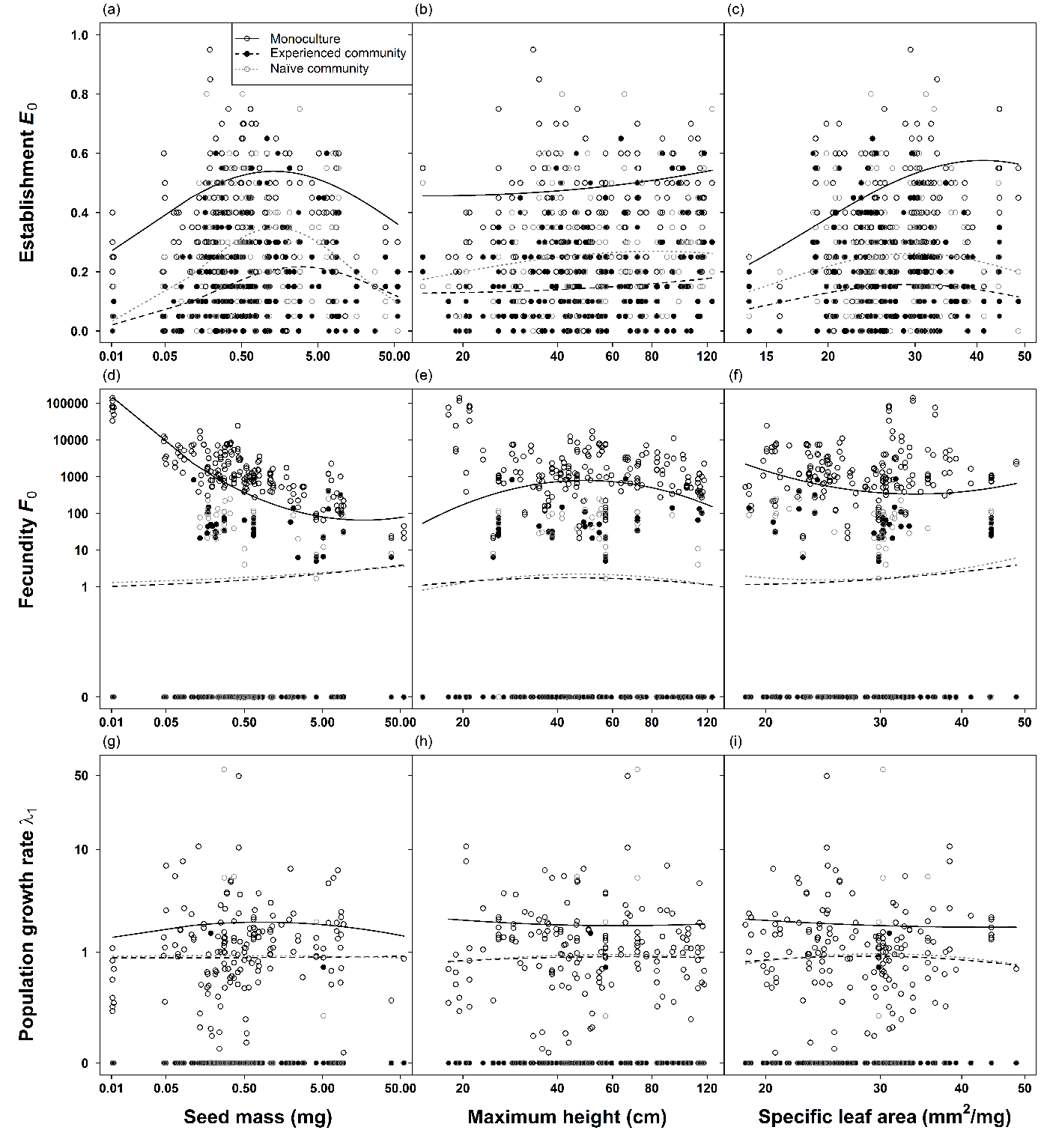


Figure S7: Relationships between functional traits (seed mass, maximum height, and specific leaf area) and (a-c) establishment (E_0_), (d-f) fecundity (F_0_) (in the first year), and (g-i) population growth rate in the second year (λ_1_) in monoculture and the two community types (experienced and naïve community). Predictions are based on the full models with the other explanatory variables set to their mean value (i.e. zero, since the functional traits were scaled and centred, allowing the response of E_0_, F_0_, and λ_1_ to any given trait to be interpreted independently of the other trait variables in the respective model). Predictions of the models contrasting monoculture vs. experienced community are shown in black (solid line: monoculture; dashed line: experienced community). Predictions for the naïve community (based on the models contrasting monoculture vs. naïve community) are shown as grey dotted line. For each demographic performance measure, both models have identical effect sizes for monoculture (see Figure S7), thus only one prediction is shown. All axes (except for E_0_) are shown on log-scale. Note that only interactions between functional traits and competition treatment in (a), (d), and (e) are significant.

**Results of analyses for effects of residence time, competition treatment, and their interaction on demographic performance**

Table S4: Properties of the models relating establishment (E_0_), fecundity (F_0_) (in the first year), and population growth rate in the second year (λ_1_) to minimum residence time, competition treatment (contrasting monoculture vs. experienced/naïve community), and their interaction.

|  |  | **Mean R^2^**  **(95% credible interval)** | | **Mean phylogenetic signal**  **(95% credible interval)** |  |
| --- | --- | --- | --- | --- | --- |
| **Demographic performance measure** | **Competition treatment** | **marginal** | **conditional** | **Pagel's lambda** | **Sample size**  **(species, populations, mesocosms)** |
| Establishment *E*_0_ | monoculture  vs.  experienced community | 0.32 (0.22, 0.41) | 0.73 (0.64, 0.83) | 0.28 (0.001, 0.57) | 40, 101, 468 |
|  | monoculture  vs.  naïve community | 0.17 (0.10, 0.24) | 0.67 (0.55, 0.79) | 0.25 (0.001, 0.53) | 40, 101, 466 |
| Fecundity *F*_0_ | monoculture  vs.  experienced community | 0.63 (0.56, 0.70) | 0.76 (0.72, 0.81) | 0.21 (0.001, 0.46) | 36, 94, 430 |
|  | monoculture  vs.  naïve community | 0.61 (0.52, 0.67) | 0.76 (0.72, 0.81) | 0.20 (0.001, 0.51) | 36, 94, 428 |
| Population growth rate λ_1_ | monoculture  vs.  experienced community | 0.41 (0.33, 0.49) | 0.62 (0.55, 0.70) | 0.15 (0.02, 0.30) | 36, 87, 394 |
|  | monoculture  vs.  naïve community | 0.32 (0.24, 0.39) | 0.52 (0.44, 0.62) | 0.05 (0.001, 0.25) | 36, 87, 392 |


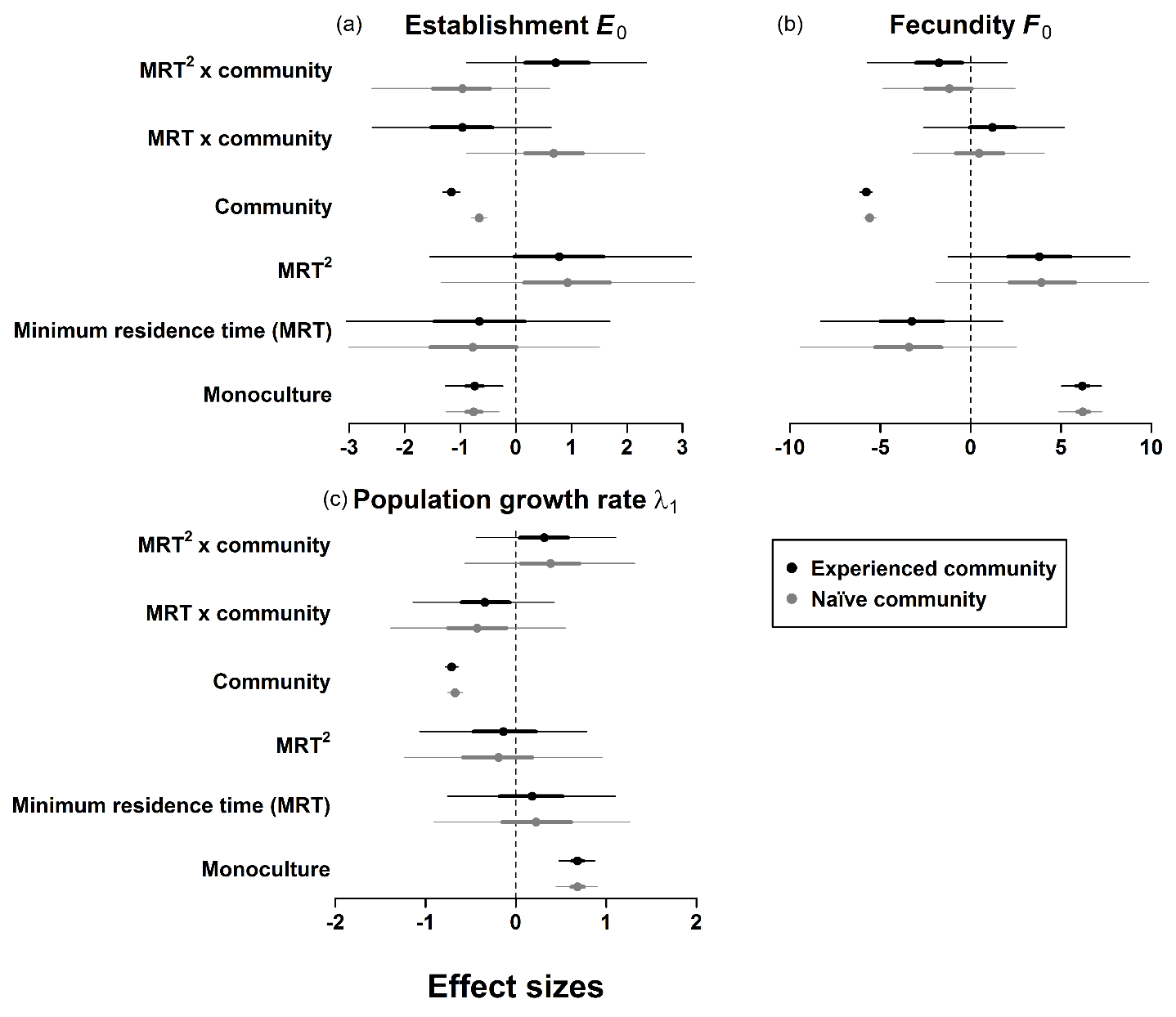


Figure S8: Effects of minimum residence time (linear and quadratic term), competition treatment (contrasting monoculture vs. experienced/naïve community, whereby monoculture represents the intercept, i.e. reference level, of the model), and their interaction on (a) establishment (*E*_0_), (b) fecundity (*F*_0_) (in the first year), and (c) population growth rate in the second year (λ_1_). Effect sizes of the models contrasting monoculture vs. experienced community are shown in black and effect sizes of the models contrasting monoculture vs. naïve community are shown in grey. Note that monoculture effect sizes refer to the intercept of the respective models and the community effect sizes refer to the contrast of monoculture vs. community. Filled circles show the posterior mean effects. Thick lines represent the 68% inner credible intervals and thin lines the 95% outer credible intervals. We consider effects to be significant, if the 95% outer credible intervals do not overlap zero.

**
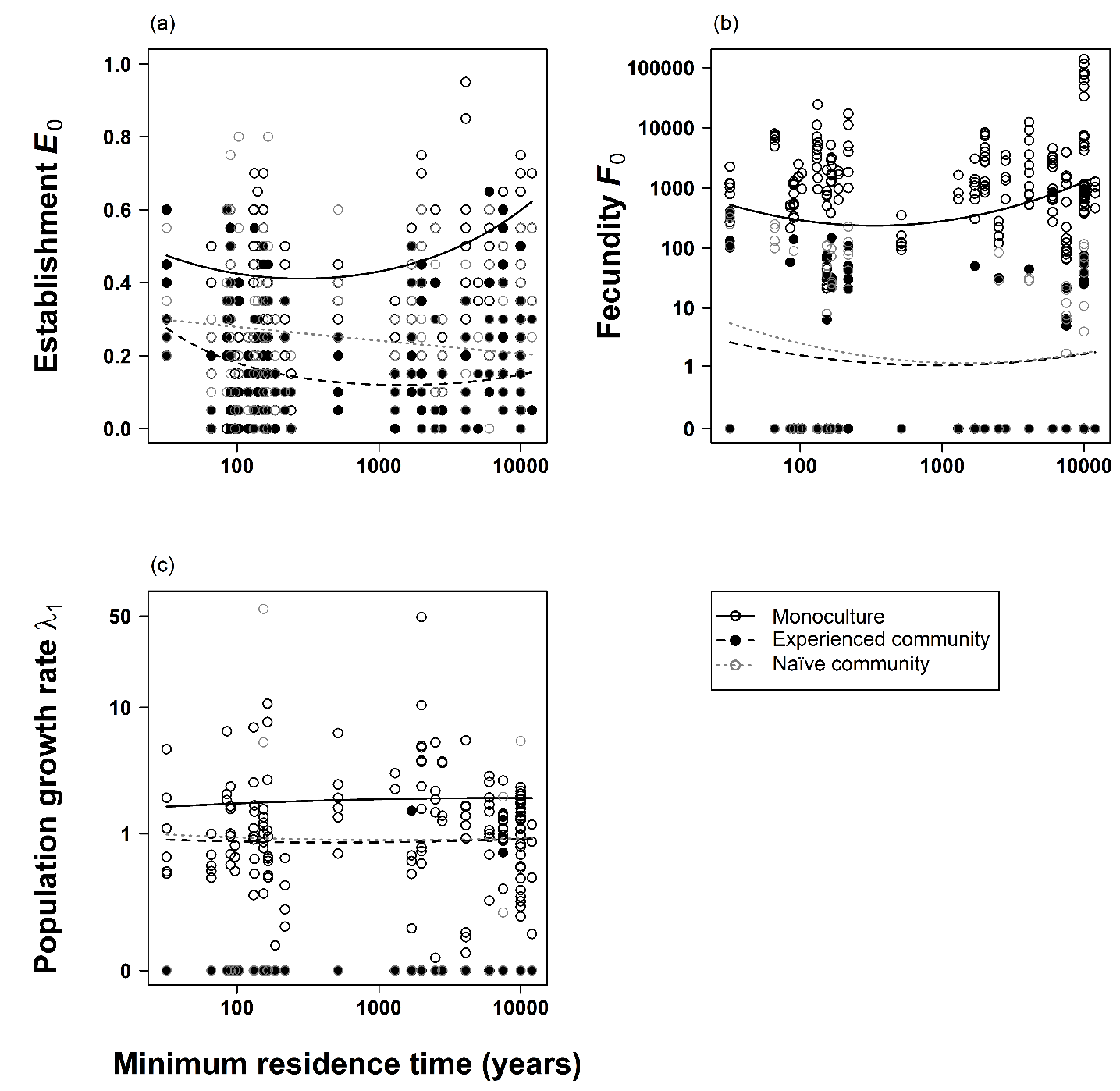
**

Figure S9: Predictions of the models relating (a) establishment (E_0_), (b) fecundity (F_0_) (in the first year), and (c) population growth rate in the second year (λ_1_) to minimum residence time, competition treatment (contrasting monoculture vs. experienced/naïve community), and their interaction. Predictions of the models contrasting monoculture vs. experienced community are shown in black (solid line: monoculture; dashed line: experienced community). The predictions of competition by the naïve community (based on the models contrasting monoculture vs. naïve community) are shown as grey dotted line. For each demographic performance measure, both models have identical effect sizes for monoculture (see Figure S5), thus only one prediction is shown. All axes (except for E_0_) are shown on log-scale.

**Control analyses for effects of functional traits, competition treatment, and residence time on fitness**

Table S5: Properties of the models for control analyses (only including wild populations) relating finite rate of increase (λ_0_) to three functional traits (linear and quadratic effect of log-transformed seed mass, maximum height and specific leaf area), competition treatment, and their interaction (top row); and to minimum residence time (MRT), competition treatment (contrasting monoculture vs. experienced/naïve community), and their interaction (bottom row).

|  |  | **Mean R^2^**  **(95% credible interval)** | | **Mean phylogenetic signal**  **(95% credible interval)** |  |
| --- | --- | --- | --- | --- | --- |
| **Model** | **Competition treatment** | **marginal** | **conditional** | **Pagel's lambda** | **Sample size**  **(species, populations, mesocosms)** |
| Functional traits | monoculture  vs.  experienced community | 0.70 (0.62, 0.77) | 0.83 (0.79, 0.87) | 0.32 (0.002, 0.53) | 24, 67, 277 |
|  | monoculture  vs.  naïve community | 0.67 (0.59, 0.75) | 0.83 (0.78, 0.86) | 0.35 (0.11, 0.61) | 24, 67, 275 |
| Minimum residence time | monoculture  vs.  experienced community | 0.63 (0.55, 0.70) | 0.77 (0.73, 0.82) | 0.29 (0.001, 0.51) | 24, 67, 277 |
|  | monoculture  vs.  naïve community | 0.61 (0.52, 0.69) | 0.78 (0.74, 0.83) | 0.31 (0.001, 0.53) | 24, 67, 275 |


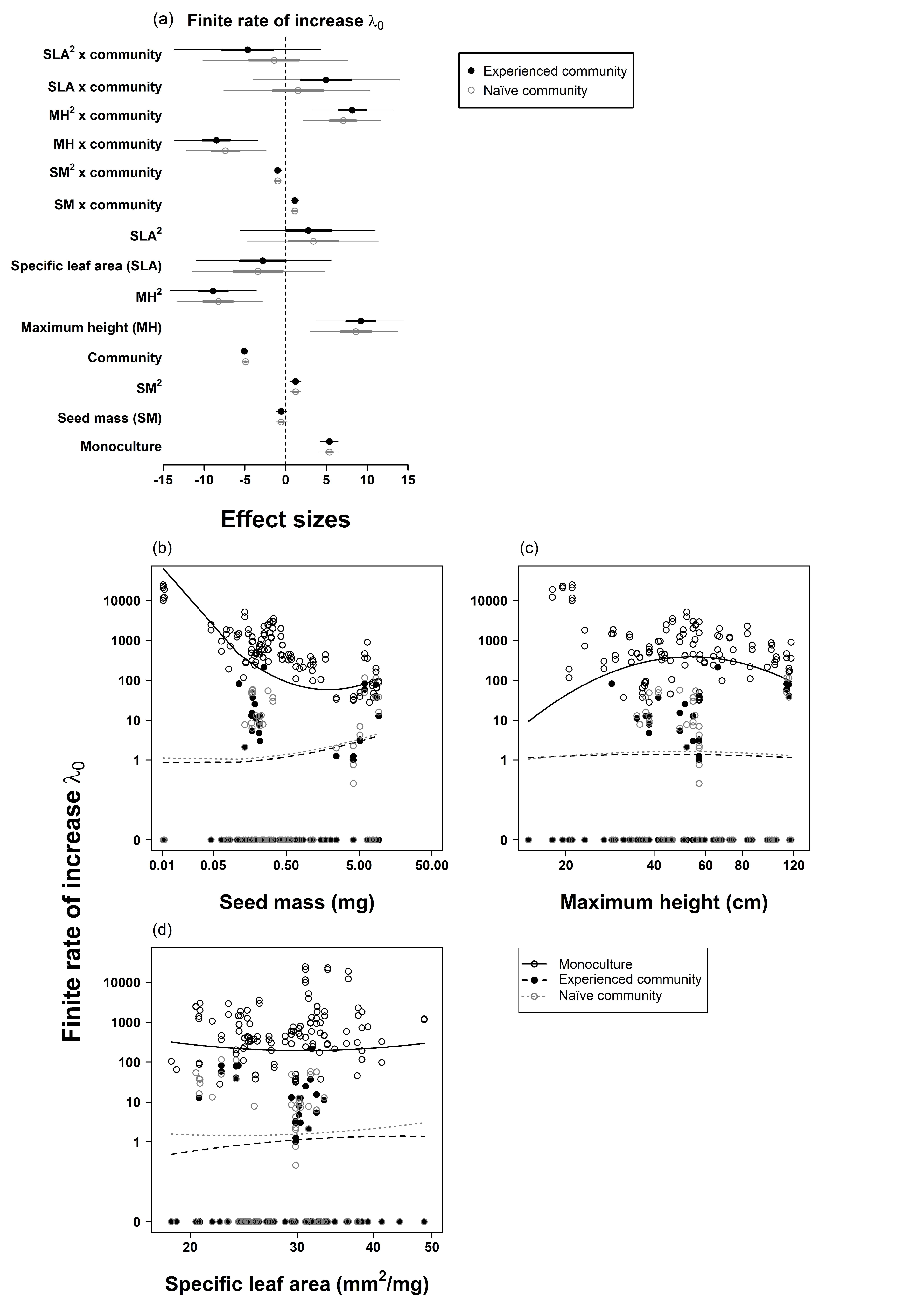


Figure S10: Effects of functional traits (linear and quadratic term of seed mass, maximum height, and specific leaf area), competition treatment (contrasting monoculture vs. experienced/naïve community, whereby monoculture represents the intercept, i.e. reference level, of the model), and their interaction on the finite rate of increase (λ_0_) only including wild populations (control analyses). (a) Effect sizes of the model contrasting monoculture vs. experienced community are shown in black and effect sizes of the model contrasting monoculture vs. naïve community are shown in grey. Note that the monoculture effect sizes refer to the intercept of the respective model and the community effect sizes refer to the contrast of monoculture vs. community. Circles show the posterior mean effects. Thick lines represent the 68% inner credible intervals and thin lines the 95% outer credible intervals. We consider effects to be significant, if the 95% outer credible intervals do not overlap zero. (b-d) Relationships between functional traits and the finite rate of increase (λ_0_) in monoculture and the two community types (experienced and naïve community). Predictions of the model contrasting monoculture vs. experienced community are shown in black (solid line: monoculture; dashed line: experienced community). Predictions for the naïve community (based on the model contrasting monoculture vs. naïve community) are shown as grey dotted line. Both models have identical effect sizes for monoculture, thus only one prediction is shown. Predictions are based on the full model with the other explanatory variables set to their mean value (i.e. zero, since the functional traits were scaled and centred, allowing the response of λ_0_ to any given trait to be interpreted independently of the other trait variables in the respective model). Note that only interactions between functional traits and competition treatment (monoculture vs. community) in (b) and (c) are significant. All axes are shown on log-scale.


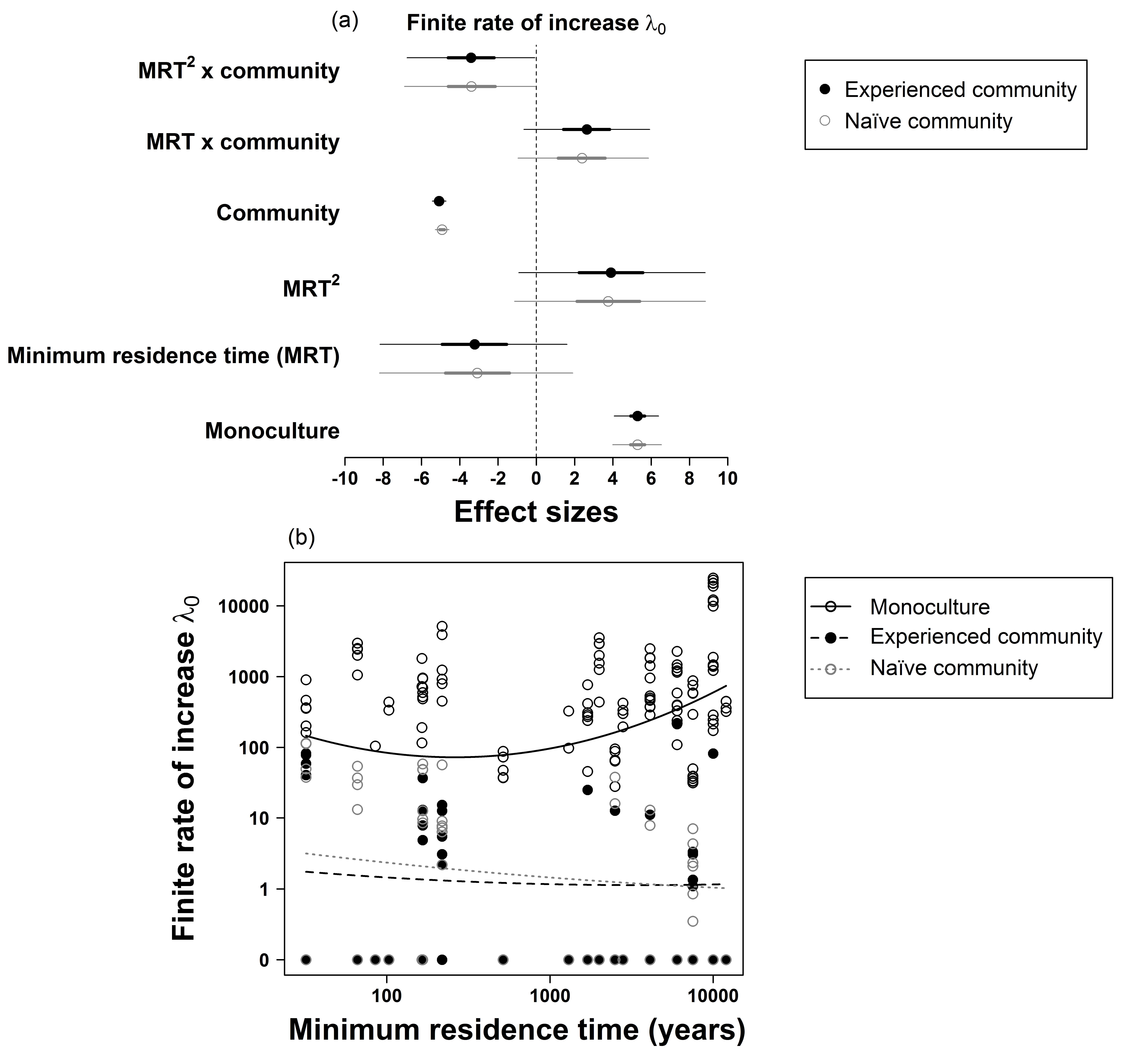


Figure S11: Effects of minimum residence time (MRT, linear and quadratic term), competition treatment (monoculture vs. experienced/naïve community, whereby monoculture represents the intercept, i.e. reference level, of the model), and their interaction on the finite rate of increase (λ_0_) only including wild populations (control analyses). (a) Effect sizes of the model contrasting monoculture vs. experienced community are shown in black and effect sizes of the model contrasting monoculture vs. naïve community are shown in grey. Note that monoculture effect sizes represent the intercept of the respective model and the community effect sizes refer to the contrast of monoculture vs. community. Circles show the posterior mean effects. Thick lines represent the 68% inner credible intervals and thin lines the 95% outer credible intervals. We consider effects to be significant, if the 95% outer credible intervals do not overlap zero. (b) Predictions of the model contrasting monoculture vs. experienced community are shown in black (solid line: monoculture; dashed line: experienced community). The prediction of competition by the naïve community (based on the model contrasting monoculture vs. naïve community) is shown as dotted grey line. Both models have identical effect sizes for monoculture, thus only one prediction is shown. All axes are shown on log-scale.

Table S6: Comparison of effect sizes (posterior mean, 95% credible interval) of the model with three levels of competition treatment (monoculture vs. experienced community vs. naïve community) to the models with two levels of competition treatment (monoculture vs. experienced community/monoculture vs. naïve community) relating finite rate of increase (λ_0_) to three functional traits (linear and quadratic effect of log-transformed seed mass, maximum height and specific leaf area), competition treatment, and their interaction.

| **Model terms** | **Model with three levels of competition treatment** | **Models with two levels of  competition treatment** | |
| --- | --- | --- | --- |
|  | **monoculture**  **vs.**  **experienced community**  **vs.**  **naïve community** | **monoculture  vs.  experienced community** | **monoculture**  **vs.**  **naïve community** |
| Monoculture (Intercept) | 5.15, 4.32 – 5.94 | 5.09, 4.14 – 6.04 | 5.10, 4.10 – 6.15 |
| Experienced community | -4.85, -5.06 – -4.61 | -4.85, -5.10 – -4.60 | / |
| Naïve community | -4.71, -4.95 – -4.50 | / | -4.71, -4.98 – -4.46 |
| Seed mass (SM) | -1.21, -1.55 – -0.84 | -1.17, -1.58 – -0.78 | -1.18, -1.57 – -0.77 |
| SM^2^ | 0.59, 0.21 – 0.96 | 0.58, 0.14 – 0.95 | 0.59, 0.20 – 1.04 |
| Maximum height (MH) | 6.14, 2.60 – 9.58 | 6.59, 2.65 – 10.20 | 6.32, 2.39 – 10.23 |
| MH^2^ | -6.02, -9.70 – -2.61 | -6.48, -10.88 – -3.22 | -6.20, -10.32 – -2.37 |
| Specific leaf area (SLA) | -6.39, -12.80 – -0.58 | -6.39, -13.78 – 0.04 | -6.66, -13.68 – 0.21 |
| SLA^2^ | 6.19, 0.40 – 12.55 | 6.23, -0.30 – 13.50 | 6.50, -0.24 – 13.60 |
| SM x experienced community | 1.40, 1.16 – 1.67 | 1.40, 1.12 – 1.66 | / |
| SM^2^ x experienced community | -0.52, -0.79 – -0.26 | -0.51, -0.84 – -0.22 | / |
| SM x naïve community | 1.37, 1.11 – 1.63 | / | 1.38, 1.10 – 1.65 |
| SM^2^ x naïve community | -0.53, -0.78 – -0.25 | / | -0.52, -0.81 – -0.21 |
| MH x experienced community | -5.75, -9.12 – -2.46 | -5.85, -9.48 – -2.12 | / |
| MH^2^ x experienced community | 5.62, 2.23 – 8.77 | 5.72, 2.09 – 9.34 | / |
| MH x naïve community | -4.46, -7.89 – -0.77 | / | -4.43, -8.13 – -0.91 |
| MH^2^ x naïve community | 4.32, 1.10 – 8.10 | / | 4.30, 0.66 – 7.71 |
| SLA x experienced community | 5.13, -1.89 – 11.75 | 5.17, -2.36 – 12.49 | / |
| SLA^2^ x experienced community | -4.86, -11.50 – 2.13 | -4.89, -12.39 – 2.58 | / |
| SLA x naïve community | 3.00, -3.82 – 9.53 | / | 2.89, -4.13 – 11.00 |
| SLA^2^ x naïve community | -2.74, -9.37 – 3.96 | / | -2.63, -10.69 – 4.48 |

Table S7: Comparison of effect sizes (posterior mean, 95% credible interval) of the model with three levels of competition treatment (monoculture vs. experienced community vs. naïve community) to the models with two levels of competition treatment (monoculture vs. experienced community/monoculture vs. naïve community) relating finite rate of increase (λ_0_) to minimum residence time (MRT), competition treatment, and their interaction.

| **Model terms** | **Model with three levels of competition treatment** | **Models with two levels of**  **competition treatment** | |
| --- | --- | --- | --- |
|  | **monoculture**  **vs.**  **experienced community**  **vs.**  **naïve community** | **monoculture  vs.  experienced community** | **monoculture**  **vs.**  **naïve community** |
| Monoculture (Intercept) | 5.14, 4.31 – 5.74 | 5.06, 3.95 – 6.02 | 5.07, 4.08 – 6.05 |
| Experienced community | -4.85, -5.12 – -4.61 | -4.86, -5.14 – -4.55 | / |
| Naïve community | -4.72, -5.10 – -4.46 | / | -4.72, -5.01 – -4.44 |
| Minimum residence time (MRT) | -3.06, -6.64 – 1.29 | -2.78, -7.14 – 1.92 | -2.81, -7.89 – 1.96 |
| MRT^2^ | 3.71, -0.25 – 7.65 | 3.46, -1.26 – 7.80 | 3.47, -1.28 – 8.61 |
| MRT x experienced community | 1.31, -1.68 – 4.31 | 1.36, -1.83 – 4.54 | / |
| MRT^2^ experienced community | -2.05, -4.93 – 1.06 | -2.12, -5.10 – 1.35 | / |
| MRT x naïve community | 0.67, -2.09 – 3.77 | / | 0.70, -2.77 – 3.97 |
| MRT^2^ naïve community | -1.51, -4.34 – 1.51 | / | -1.53, -5.12 – 1.65 |
